# Supplementary material for: Direct cysteine sulfenylation drives activation of the Src kinase
Source: Nat Commun. 2018 Oct 30;9:4522. doi: 10.1038/s41467-018-06790-1 (PMC6207713; doi:10.1038/s41467-018-06790-1)
Supplement: Supplementary file 1 — Supplementary Information [file 41467_2018_6790_MOESM1_ESM.pdf]

Direct Cysteine Sulfenylation Drives Activation of the Src Kinase

Heppner, Dustin, and Liao et al.,

Supplementary Methods

Experimental Methods

Recombinant Src expression and purification. The plasmid pEX-Src-C-His (Origene, Rockville, MD and Blue Heron Biotech, Bothell, WA) was used to transform BL21-AI *E. coli* (Thermo). The C185A and C277A mutants were generated from the pEX-Src-C-His plasmid with the QuickChange Mutagenesis kit (Agilent Technologies, California) with the following primers:

C185A Forward:	CGAAAGGTGCCTACGCCCTCTCAGTGTCTGAC
C185A Reverse:	GTCAGACACTGAGAGGGCGTAGGCACCTTTCG
C277A Forward:	CTGGGCCAGGGCGCCTTTGGCGAGGTG
C277A Reverse:	CACCTCGCCAAAGGCGCCCTGGCCCAG

Colonies containing the mutated plasmid DNA were selected on Ampicillin plates, grown in 5 mL cultures (LB with ampicillin), and the plasmid DNA was isolated using the Zippy Plasmid Miniprep kit (Zymo Research, Irvine, CA). Each mutation was confirmed by DNA sequencing using the following sequencing primers: (GGACTTTCCAAAATGTCG; ATTAGGACAAGGCTGGTGGG; ATTGCTCAGCGGTTATCGTG). The mutated plasmid DNA was then transformed into BL21-AI cells and colonies were selected for subsequent growth in the expression studies.

BL21-AI *E. Coli* (ThermoFisher) containing the pEX-Src-C-His vector was grown overnight at 37 ºC in LB broth (Invitrogen) supplemented with 100 µg/mL ampicillin (Fisher). The following day, cultures were pelleted and resuspended in fresh LB broth for inoculation of TB broth (Sigma) supplemented with 100 µg/mL ampicillin. TB cultures were further incubated at 37 ºC until an optical density of 0.6-1.2 was reached (~ 3 hrs). Cultures were cooled and supplemented with 1 mM isopropyl β-D-1-thiogalactopyranoside and 0.2% arabinose to induce expression, followed by overnight incubation at 18 ºC. Cells were pelleted and stored frozen at -20ºC.

Recombinant Src-His tagged protein was purified using a modified version of the protocol described previously[1]. Frozen pellets were thawed and resuspended in 4 mL Src A buffer (50 mM Tris, 500 mM NaCl, 25 mM Imidazole, pH 8.0) per gram of cell paste and lysed via sonication. Lysate was treated with DNAse I (Qiagen) to reduce viscosity for 10 minutes prior to centrifugation. The lysate supernatant was then diluted twofold with buffer and loaded on a chelating sepharose column (GE Life Science) preloaded with Nickel Sulfate (Sigma) and equilibrated to Src A buffer. The bound protein was washed with Src A buffer until the flow-through showed minimal absorbance at 280 nm (less than 0.05 AU). Protein was then eluted with Src B buffer (50 mM Tris, 500 mM NaCl, 500 mM Imidazole, pH 8.0). Fractions were analyzed for protein content using 10% SDS-PAGE with Coomassie Brilliant Blue stain.

Positive fractions were pooled and supplemented with 1 mM DTT, followed by overnight dialysis against QA buffer (25 mM Tris, pH 8.0) using Slide-a-Lyzer dialysis cassettes (Thermo). The following day, protein was collected and supplemented with 1 mM DTT. Protein was then

loaded on a Q-sepharose column (GE Life Science) pre-equilibrated with QA buffer containing 1 mM DTT and washed with QA buffer + DTT until the flow-through showed minimal absorbance at 280 nm. Src was then eluted from the column with a 400 mL gradient of 0.0-1.0 M NaCl. Protein was detected using Bradford assay and SDS-PAGE. Positive fractions were pooled and buffer exchanged into storage buffer (50 mM Tris, 100 mM NaCl, 5% glycerol, 1 mM DTT, pH 8.0) and quantified with the Bradford assay. Stock solutions were kept at 1-3 mg/mL and stored at -20ºC.

***Analysis of Src Oxidation by Mass Spectrometry*.**

<u>Sample preparation.</u> For global sulfenylation analysis, recombinant Src (~20 µg for dimedone experiments; ~13 µg for BCN experiments) buffered in 100 mM Tris-HCl, 20 mM $MgCl_2$, 2 mM EGTA, 0.02% Brij-35, 1.0 mM DTT at pH 7.4 was preincubated with either 1.0 mM 5,5'-dimethyl-1,3-cyclohexanedione (dimedone; Sigma) or with 100 µM 9-hydroxymethylbicyclo[6.1.0.]nonyne (BCN, Sigma) for 10 min and then reacted with H_2O_2 (Sigma) for 1 hr at room temperature in a total volume of 30 µL. The final DTT concentration in these experiments was ~0.25 mM in the BCN studies and ~0.03 mM in the dimedone studies. In separate analyses, Src was also reacted with H_2O_2 simultaneously with 5 mM 4-chloro-7-nitrobenzofuran (NBD-Cl), which modifies reduced thiols (Cys-SH) as well as sulfenic acids to generate adducts with +16 mass difference[2, 3] and reacted for 1 hr at 25°C. For quantitative analysis of dimedone labeling by targeted mass spectrometry with parallel reaction monitoring (PRM), 20 µg of recombinant Src was treated with 0 mM H_2O_2 ("non-oxidized") in the presence of dimedone (dim-d_0), and analogous samples were treated with 1.0 mM H_2O_2 ("oxidized") in the presence of the deuterium-containing variant of dimedone (dim-d_6; Kerafast). Prior to SDS-PAGE desalting and gel extraction, excess H_2O_2 was quenched with 1 µL of 42,000 U/mL catalase and equal amounts of d_0 and d_6 samples were mixed.

Protein samples were separated on 10% SDS-PAGE gels and visualized by Coomassie staining. Bands positive for Src (~60 kDa) were excised and transferred to clean microcentrifuge tubes. Gel pieces were then washed with 250 µl H_2O for 15 min and destained with 50 mM ammonium bicarbonate in 50% acetonitrile. Following complete removal of coomassie stain, gel pieces were washed with 500 µl H_2O. Pieces were then dehydrated using 100% CH_3CN, which was removed using a speed vac (Labconco) prior to reduction with 200 µl 10 mM DTT in 100 mM NH_4HCO_3 for 1 h at 56°C. Free Cys residues were then alkylated by subsequent incubation with 55 mM iodoacetamide in 100 mM NH_4HCO_3 in the dark for 45 min at room temperature. After removal of iodoacetamide, gel pieces were washed with 100 mM NH_4HCO_3 and dehydrated with 100 µl CH_3CN for 10 min, which was repeated once, after which dehydrated gel pieces were dried in a Speed Vac, and re-swelled with 12 ng/µL trypsin (Promega V511A, 5–20 µg/ml; to achieve trypsin:protein ratio of 1:20 to 1:100 w/w) at 4 °C for 30 min and subsequently incubated overnight at 37 °C for complete protein digestion. Peptides were extracted with sequential treatments of 5% formic acid (FA) in H_2O, 5% FA in 50% acetonitrile, and finally 100% acetonitrile. Extracted peptide solution was then Speed-vac dried and kept at -20°C. Dried peptides were reconstituted with 2.5% CH_3CN/2.5% formic acid for LC–MS/MS analysis.

<u>MS analyses.</u> Samples from BCN-related experiments were analyzed by capillary LC–MS/MS on a Q-Exactive mass spectrometer coupled to an EASY-nLC (Thermo Fisher Scientific, Waltham, MA, USA). The samples were loaded onto a 100 µm x 120 mm capillary column packed with

Halo C18 (2.7 µm particle size, 90 nm pore size, Michrom Bioresources, CA, USA) at a flow rate of 300 nl min^{-1}. Peptides were separated using a gradient of 2.5-35% CH_3CN/0.1% FA over 150 min, 35-100% CH_3CN/0.1% FA in 1 min and then 100% CH_3CN/0.1% FA for 8 min, followed by an immediate return to 2.5% CH_3CN/0.1% FA and an isocratic hold at 2.5% CH_3CN/0.1% FA until the next injection. Peptides were introduced into the mass spectrometer via a nanospray ionization source and a laser pulled ~3 µm orifice with a spray voltage of 2.0 kV. Mass spectrometry data was acquired in a data-dependent "Top 10" acquisition mode with lock mass function activated (*m/z* 371.1012; use lock masses: best; lock mass injection: full MS), in which a survey scan from *m/z* 350-1600 at 70,000 resolution (AGC target $1e^6$; max IT 100 ms; profile mode) was followed by 10 higher-energy collisional dissociation (HCD) tandem mass spectrometry MS/MS scans on the most abundant ions at 17,500 resolution (AGC target $5e^4$; max IT 100 ms; centroid mode). MS/MS scans were acquired with an isolation width of 1.6 *m/z* and a normalized collisional energy of 26%. Dynamic exclusion was enabled (peptide match: preferred; exclude isotopes: on; underfill ratio: 1%; exclusion duration: 30 sec).

Samples from dimedone and NBD-Cl experiments were analyzed on a linear ion trap (LTQ) mass spectrometer coupled to a Surveyor MS Pump Plus (Thermo Fisher Scientific, MA). Half of the digest was loaded directly onto a 100 µm×120 mm capillary column packed with MAGIC C18 (5 µm particle size, 20 nm pore size, Michrom Bioresources, CA) at a flow rate of 500 nL/min, and peptides were separated by a gradient of 3–35% CH_3CN/0.1% FA over 45 min, 35–100% CH_3CN/0.1% FA in 1 min, and 100% CH_3CN/0.1% FA in 9.5 min. Peptides were introduced into the linear ion trap via a nanospray ionization source and a laser pulled ~3 µm orifice with a spray voltage of 1.8 kV. Mass spectrometry data were acquired in a data-dependent "Top 10" acquisition mode, in which a survey scan from *m/z* 400 -2000 is followed by 10 collision-induced dissociation (CID) tandem mass spectrometry MS/MS scans of the most abundant ions. MS/MS scans were acquired with the following parameters: isolation width: 2 *m/z*, normalized collision energy: 35%, Activation Q: 0.250 and activation time = 30 ms. Dynamic exclusion was enabled (repeat count: 2; repeat duration: 30 sec; exclusion list size: 500; exclusion duration: 60 sec). The minimum threshold was 500.

Product ion spectra were searched using the SEQUEST search engine implemented on the Proteome Discoverer 1.4 (Thermo Fisher Scientific, Waltham, MA, USA) against a curated Uniprot *homo sapiens* database (downloaded on Feb 17 2015) in forward and reverse orientations. Search parameters were as follows: (1) full trypsin enzymatic activity, (2)~~,~~ maximum missed cleavages = 2, (3) min. peptides length = 6, (4) Mass tolerance: LTQ: 2 Da for precursor ions and 0.8 Da for fragment ions, Q-Exactive: 20 ppm for precursor ions and 0.02 Da for fragment ions (5) Dynamic modifications on cysteine (BCN / +166.099 Da, Dimedone / +138.068 Da, Oxidation / +15.9949 Da, Dioxidation / +31.990 Da, Trioxidation / +47.985 Da, Carbamidomethylation / +57.0215 Da, NBD-Sulfoxide (SO-NBD) / +178.997 Da, NBD-thioether (S-NBD)/ +163.002 Da) and (6) 4 maximum dynamic modifications allowed per peptide. False positive rates were limited to less than 1% in the data sets by including Percolator node in the workflow.[4] The search results were further analyzed using Scaffold 4.05 (Proteome Software, OR) for sequence annotation.

For targeted mass spectrometry experiments involving quantification of dimedone-d0 and dimedone-d6 labeled peptides, mass spectrometry data was acquired with alternating MS-SIM scans and PRM (2 scan groups in the method). Full scans were acquired from *m/z* 340 - 1,400 at

70, 000 resolution (AGC target $3e^6$; max IT 200 ms; profile mode). PRM were carried out with HCD MS/MS scans at 17,500 resolution on the precursors imported into the inclusion list, with the following settings: AGC target $2\ e^5$; max IT 100 ms; isolation width of 1.2 *m/z* and a normalized collisional energy of 28%. The dimedone and dimedone-d_6 labeled Src peptide pairs with various charge states (CS) were monitored [AANILVGENLVCK (CS: 2; *m/z:* 741.4053, 744.4241); AANILVGENLVC(dim/dim-d6)KVADFGLAR (CS: 3; *m/z:* 771.0875, 773.1000); GAYC(dim/dim-d6)LSVSDFDNAK (CS: 2; *m/z*: 814.3691, 817.3879); HADGLC(dim/dim-d6)HR (CS: 3; *m/z:* 351.5120, 349.4994); LGQGC(dim/dim-d6)FGEVWMGTWNGTTR (CS: 2; *m/z:* 1119.5090, 1122.5279); LTTVC(dim/dim-d6)PTSKPQTQGLAK (CS: 3; *m/z:* 637.6801,639.6926); MPCPPECPESLHDLMCQCWR (with 1 dim/dim-d6 modified cysteine and 3 carbamidomethylated cysteines; CS: 2; *m/z:* 1342.5483, 1345.5672)]

MS/MS spectra were searched against the Src sequence using SEQUEST on Proteome Discoverer. For the quantification experiments involving dimedone/dimedone-d6, the search files (.msf) were then imported into Skyline for selecting the precursor or transitions for quantitation. Data was exported from XCalibur and Skyline to GraphPad Prism 7 for chromatogram/spectrum plotting. Four to six transitions were selected from, if possible, the third to the last fragment ions in the product ion spectrum for quantification. Boundaries of integration were manually evaluated. For MPC(dim/dim-d6)PPECPESLHDLMCQCWR (the other 3 cysteines were carbamidomethylated), its quantification was performed with transitions 1342.5483 m/z $\rightarrow$ [y3]+, [y7]+, and [y13]+ (dim labeled) and 1345.5672 m/z $\rightarrow$ [y3]+, [y7]+, [y13]+ (dim-d6 labeled) using peak integration in XCalibur. Data was exported from XCalibur and Skyline to GraphPad Prism 7 for plotting chromatograms and spectra.

Tyrosine kinase activity measurements. Tyrosine kinase activity of recombinant Src and Cys-to-Ala variants was analyzed using the ADP-Glo (Promega) assay kit according to the manufacturer's protocol in kinase buffer containing 2.0 mM DTT. WT, C185A, and C277A Src (~1 ng) was pre-treated at room temperature with hydrogen peroxide (H_2O_2; Thermo) for 15 min in a volume of 15 µL, prior to initiating catalysis with substrate (poly[4Glu:Tyr]; Sigma) and ATP (Promega), resulting in a final reaction volume of 25 µL, for 60 min at room temperature within the linear kinetic range (data not shown). Reactions were quenched with ADP Glo reagent for 40 min, further incubated with detection reagent for 30 mins, and measured for luminescence. Specific activity (µmol ATP/min/µg protein) was obtained from an ADP (Promega) standard curve.

Analysis of disulfide cross-linking by non-reducing SDS-PAGE. To assess the capability of Src to form disulfide bonds, recombinant Src (10 µg) buffered in 100 mM Tris-HCl, 20 mM $MgCl_2$, 2 mM EGTA, 0.02% Brij-35, 1.0 mM DTT at pH 7.4 was incubated with increasing concentrations of H_2O_2 at room temperature for 1h. Following 1h incubation, protein was treated either with 100 mM DTT or with water for 30 min, followed by the addition of non-reducing laemmli buffer. Samples were boiled at 100°C for 5 minutes prior to SDS-PAGE, and proteins were visualized using coomassie blue staining.

Cell culture and treatments. Human pulmonary mucoepidermoid NCI-H292 cells (American Type Culture Collection) were grown in RPMI 1640 medium containing 10% fetal bovine serum and 1% penicillin/streptomycin at 37°C and 5% CO_2. Src-FLAG constructs were generated by

transiently transfecting H292 cells at 85-90% confluence using the Turbofect reagent (Thermo) according to a modified version of the manufacturer's protocol. 1 µg of corresponding pCMV6-Src (Origene) was mixed with 8 µL Turbofect and 100 µL serum-free RPMI media (per well) and allowed to incubate at RT for 20 min. Cells were changed to 900 µL serum-free media prior to dropwise addition of 100 µL transfection mix in each well. Cells were then incubated at 37 °C for 24 hrs to allow for complete DNA transfection, followed by PBS washing to remove any cells that may have died during the process. Following transfection, cells were grown again for 24 hrs with full RPMI media to allow for regeneration. Prior to subsequent experiments (generally ~48-72 hrs post-transfection, depending on visual assessment of viability), cells were switched to serum-free RPMI for overnight incubation. Stable H292 cell lines expressing C-terminal FLAG-tagged WT Src, C185A, and C277A, were generated by transfecting ~70% confluent cells in a 24-well dish (Corning) with 1 µg of DNA from the pCMV6-Src (RC208622 Origene Rockville, MD) and 2 µL of Turbofect Transfection reagent (Fisher) in serum free media for 24 hrs as per manufacture's protocol. Mutant plasmids were generated using identical methods and primers as mentioned above with the pEX-Src-C-His vector. Cells were then washed with PBS (Gibco) and then cultured for 24 hrs with serum containing media. Cells were then selected over 10-15 days in serum containing media with 150 µg/mL of neomycin (G418 sulfate). Successful transfection was confirmed on the basis of Western blot for FLAG-tagged Src at a molecular weight of ~65 kDa (Figure S8). For experimentation, cells were seeded at 100,000 cells/well in 24-well plates (Corning). Upon reaching confluence, cells were cultured overnight in the absence of serum and placed in fresh serum-free medium for 1-2 hrs before stimulation with exogenous ATP (Sigma, St. Louis, MO; 100 µM) for 10 minutes.

Western blotting. Cell lysates were collected by placing cells on ice in 100 µL Western solubilization lysis buffer (50 mM HEPES, 250 mM NaCl, 1.5 mM $MgCl_2$, 1% Triton-X100, 10% glycerol, 1 mM ethyleneglycol-bis-(β-aminoethylether)-*N*,*N*,N^9,N^9-tetraacetic acid, 1 mM phenylmethylsulfonyl fluoride, 2 mM Na_3VO_4, 10 mg/mL aprotinin, and 10 mg/mL leupeptin; pH 7.4) per well for 30 min. Lysates were collected by scraping, briefly sonicated, and cleared of insoluble material by centrifugation (14,000 rpm, 5 min) for analysis. Lysates containing equal amounts of protein (15–35 µg, measured using BCA protein assay kit; Pierce) were loaded on 10% SDS-PAGE gels and transferred to nitrocellulose membranes, and probed using antibodies against p-Src Tyr 416 (1:400; 2101S), p-Src Tyr 527 (1:400; 2105S), Src (L4A1; 1:1000; 2110S), FLAG (DDK; 1:1000; 2368S) (Cell Signaling), or streptavidin peroxidase polymer ultrasensitive (1:10,000; S2438; Sigma). Primary antibodies were probed with rabbit or mouse-specific secondary antibodies conjugated with HRP (Cell Signaling) and detected by enhanced chemiluminescence (Pierce). Western blot band densities were quantified using ImageQuant TL (v8.1.0.0).

Analysis of protein sulfenylation. For analysis of protein sulfenylation (-SOH), cells were lysed in Western solubilization buffer containing 1 mM DCP-bio1 (Kerafast or EMD Millipore), 200 U/mL catalase (Worthington, Lakewood, NJ) and 10 mM *N*-ethylmaleimide (Sigma) and incubated for 1 hr on ice. FLAG-tagged WT, C185A, and C277A Src was purified from cell lysates (~300 µg for WT, C185A and ~900 µg for C277A) with ~40 µL packed volume of Anti-FLAG M2 magnetic beads (Sigma) as per manufacture's protocol. M2 beads were washed 5 times with 1 mL of 50 mM Tris and 150 mM NaCl pH 7.4 and collected using a DynaMag2 magnetic particle separator. FLAG-tagged proteins were eluted with 2x washes of Tris buffer

containing 150 µg/mL 3X FLAG peptide (Sigma). Eluted proteins were combined with sample buffer and run on 10% SDS PAGE gels. Additionally, to further probe for *in situ* sulfenylation, cells were pre-loaded with 5 mM DYn-2 (Kerafast) for 15-30 min prior to ATP stimulation. Cells were then lysed with HEPES lysis buffer (50 mM Hepes, 150 mM NaCl, 1% NP-40, 0.1% SDS, pH 7.4) supplemented with 10 mM NEM, Halt protease and phosphatase inhibitor cocktail (no added EDTA, Thermo), and 200 U/mL catalase. ~100 ug of protein lysate was the pre-cleared of endogenously biotinylated proteins with NeutrAvidin agarose resin for 1 hr prior to incubation with a 1:1 ratio click reaction mix (200µM Biotin-Azide (Kerafast), 500 µM $CuSO_4$, 1 mM Tris[(1-benzyl-1*H*-1,2,3-triazol-4-yl)methyl]amine (TBTA, Sigma), and 5 mM Sodium L-ascorbate. pH 7.0) for 1h with gentle agitation. This reaction was then quenched with the addition of 1 mM EDTA and analyzed in the same manner as DCP-Bio1 conjugated samples.

Immunofluorescence. H292 cells stably expressing either FLAG-Src WT or pCMV6 (Origene, Rockville, MD) empty vector controls were seeded on Millicell 8-well glass chamber slides (Millipore, Billerica, MA) at a density of 75,000 cells/well and grown to ~95-100% confluence. Cells were serum starved overnight prior to washing with PBS, fixation with 4% Paraformaldehyde, and permeabilization with 0.2% Triton X-100 in a 1% BSA/PBS solution for 15 min. Cells were washed prior to blocking with 10% normal goat serum (Life Technologies, Carlsbad, CA) for 1h. After blocking, cells were then washed prior to overnight incubation at 4°C with α-Src (36D10 rabbit, 1:500, Cell Signaling Technologies, Danvers, MA) and M2 α-FLAG (F1804 mouse, 1:500, Sigma, St. Louis, MO) antibodies. The following day, cells were washed prior to incubation with AlexaFluor-conjugated α-rabbit (A-21245 AlexaFluor 647, 1:500, Life Technologies, Carlsbad, CA) and α-mouse (A-21422 AlexaFluor 555, 1:500, Invitrogen, Carlsbad, CA) secondary antibodies. Cells were then washed, followed by nuclear counterstaining with 10 µg/mL 4, 6-diamidino-2-phenylindole (DAPI, Invitrogen, Carlsbad, CA) for 15 min in 1% BSA. Finally, cells were washed and a glass coverslip was mounted to the slide. Images were collected using a Zeiss LSM 510 META laser scanning confocal microscope (Zeiss, Jena, Germany).

Molecular dynamics simulations

<u>Model Preparation.</u> The protein models were constructed from the autoinhibited Src structure (PDB: 2SRC)[5]. The models containing sulfenic acid cysteines at C185, C277, and C498 were constructed using Maestro (Schrödinger *Inc.*). Since both Cys-SH and Cys-SOH are weak acids, they could potentially be present in their deprotonated forms. We therefore calculated pK_a values of simple CH_3CH_2-SH and CH_3CH_2-SOH models using pK_a calculations in Jaguar (Schrödinger *Inc.*) with DFT method (B3LYP, pka-geopt* basis, water solvent), which revealed pK_a values of 10.3 for the thiol and 8.7 for the sulfenic acid form, and therefore performed MD simulations based on protonated states of Cys-SH and Cys-SOH. A web-based graphical user interface CHARMM-GUI[6] was used to prepare the initial models for simulations, which contain our protein models, 17,000~26,000 TIP3P water molecules, chloride potassium as counter ions, totaling near 58,400~85,400 atoms in a periodic box 85 × 89 × 82~94 × 94 × 94 Å^3. The parameters (i.e. for bonds, angles, and dihedral angles) and partial charges for cysteine sulfenic acid, which were initially generated using CHARMM General Force Field (CGenFF)[7] in CHARMM-GUI[6], were validated or modified using quantum calculations at the density functional theory (DFT) level (see ***Cysteine sulfenic acid force field parameters*** for

parametrization). Since both Cys-SH and Cys-SOH are weak acids, they could potentially be present in their deprotonated forms. We therefore calculated pK_a values of simple CH_3CH_2-SH and CH_3CH_2-SOH models using pK_a calculations in Jaguar (Schrödinger, Inc.) with DFT method (B3LYP, pka-geopt* basis, water solvent), which revealed pK_a values of 10.3 for the thiol and 8.7 for the sulfenic acid form, and therefore performed MD simulations based on protonated states of Cys-SH and Cys-SOH.

Simulation Setup. We set up two replica simulations for each system in Table S2. All simulations were performed with the CHARMM36 force field[8] in addition to our customized parameters for the Cysteine Sulfenic Acid (Cys-SOH). For each construct, we employed an equilibrium strategy including stages of minimization, equilibration, and a short simulation (for 10 ns) in the NAMD[9] program. After equilibrium, the long simulations (for 1-5 µs) were carried out on the specialized ANTON supercomputer using the software program Anton 2.13.0[10] and with the Desmond program on GPUs (Schrödinger, Inc.). All our simulations were performed with the NPT ensemble (300 K, 1 bar, Nose-Hoover coupling scheme). The van der Waals and short-range electrostatics were cut off at 12.0 Å with a switch at 10.0 Å. Hydrogen atoms were constrained using the SHAKE algorithm. A summary of our simulations is provided in Table S2.

Metadynamics simulations. Metadynamics simulations were carried out to determine the two-dimensional free-energy landscapes of conformational transition in the cysteine oxidation region in comparison with it of wild type. Metadynamics simulations were setup in Desmond (Schrödinger, *Inc.*). By adding a time-dependent bias in the form of repulsive Gaussians as a function of carefully chosen collective variables (CVs), the system is coaxed to escape stable free energy minima where it would normally be trapped.[11] According to the key residue interactions in MD simulations, two collective variables (CVs) were chosen to characterize the conformational change around each cysteine oxidation region as following. (1) The pair distances of Y416-D386 (CV1) and R419-D386 (CV2) are used to characterize the dissociation of A-loop in the Cys-277 system (Figure 4). (2) The pair distances of pY527-R175 (CV1) and pY527-R155 (CV2) are used to characterize the dissociation of pY527 from SH2 region in the Cys-185 system (Figure 5). (3) The center-of-mass distances of helix 489-499 to helix 446-457 (CV1) and center of mass distances of helix 489-499 to helix 360-371 (CV2) are used to characterize the dissociation of helix 489-499 from the helices bundle near the C-loop in the Cys-498 system (Figure S14). The upper boundary of all CVs are within the range of 20~25 Å. The gaussians width is set as 0.05. To examine the convergence of free-energy landscape, we have calculated the free-energy difference every 10 ns after 80 ns: the key energy basins remain to the final 120 ns with a general standard deviation of 0~0.68 kcal/mol for the maximum free-energy difference.

Simulation Data Analysis. Our simulation data analysis includes helicity percentage and solvent contacts, which were performed with TCL scripts implemented in VMD 1.9.2[12] and plotted by Prism (version 7.0a). Visualizations were performed with the programs VMD[12] and Pymol (Schrödinger, Inc.). Helicity percentage was calculated for residues 406-423. Solvent contacts were calculated by counting the number of water molecules around pTyr-527 within radius of 6 Å.

Cysteine sulfenic acid force field parameters. The CHARMM36 force field parameters for the cysteine sulfenic acid, which include the structural parameters (i.e. for bonds, angles, and dihedral angles) and partial charges, were initially generated using CGenFF[7] in CHARMM-GUI[6]. These parameters have been validated or modified according to DFT optimized models. To obtain partial charges for the cysteine sulfenic acid, a model ($^{+}H_3N$-C(CH_2SOH)-COO^{-} was constructed in Maestro and several representative conformations were obtained from a Low-Mode Conformational Search using MacroModel BatchMin v10.4). Representative partial atomic charges from electrostatic potentials (ESP) were obtained from single point calculations on the resulting conformation using DFT with B3LYP/6-31g** in Jaguar v8.4 [13, 14]. Van der Waals parameters were set to those for the disulfide (-S-S-) sulfur atoms since the sulfenic acid oxidation state is similar to that of a disulfide. The bond-stretching and angle-bending parameters obtained from CGenFF[7] are reliable with penalties below 10, while the parameters for dihedral angle –C-S-O-H we were to apply to the CHARMM force filed needed further validation. Therefore, we performed relaxed scan for dihedral angles using the program Jaguar (Schrödinger, *Inc.*). In a relaxed scan, the structure will be optimized at each scan point.

We used the CH_3CH_2-SOH model system to compare our parameters for -SOH with DFT calculations geometrically and energetically in dihedral angle scanning. We scanned the dihedral angle –C-S-O-H from 0 degrees to 180 degrees, over 10-degree increments from point to point in implicit water using the Poisson-Boltzmann model. At each scan point, a series of single-point energy calculations using DFT with B3LYP/6-31g** was applied while geometry optimization was also carried out, which is a general force-filed parameterization scheme.[15] The total energy difference by relaxed dihedral scanning using DFT calculations was displayed in blue line in Figure S11.

The dihedral term in the CHARMM force filed was given by,

$$V(\phi) = K_\phi[1 + \cos(n\phi - \phi_0)] \qquad (Supplementary\ Equation\ 1)$$

K_ϕ is the dihedral force constant, n is the multiplicity of the function, ϕ_0 is an equilibrium angle. The best fitting dihedral parameter is: K_ϕ, n, and ϕ_0 values as 3.2, 2, and 0, respectively. Here, we chose K_ϕ of 1.1, referring to other value given to similar groups used in the CHARMM force filed, for example, -C-C-O-H; the less energy barrier may contribute to faster dynamics and easier rotation for–C-S-O-H, within conformational error range. The dihedral term energy calculated from supplementary equation 1 is shown in orange line in Supplementary Fig. 11, which generated standard deviation of 2.2 kcal/mol. Accordingly, the total energy difference of each structure generated from Jaguar's relax-scanning were calculated in NAMD program and displayed in red line in Supplementary Fig. 11. Generally, our dihedral-angle-dependent energy profile is consistent with DFT calculations, which validate the dihedral angle geometry preference for the sulfenic acid group (-SOH). The cysteine sulfenic acid force field parameters are displayed below.

>>>Topology File Parameters

```
RESI SOH         0.00
GROUP
```

```
ATOM N    NH1     -0.47  !      |
ATOM HN   H        0.31  !  HN-N
ATOM CA   CT1      0.07  !      |   HB1
ATOM HA   HB1      0.09  !      |   |
GROUP                    !  HA-CA--CB--SG
ATOM CB   CT2     -0.20  !      |   |      \
ATOM HB1  HA2      0.14  !      |   HB2    O1--H1
ATOM HB2  HA2      0.14  !    O=C
ATOM SG   SO1     -0.05  !      |
ATOM O1   OH1     -0.43
ATOM H1   H        0.40
GROUP
ATOM C    C        0.51
ATOM O    O       -0.51
BOND CB CA   SG CB   N HN  N  CA
BOND C  CA   C +N  CA HA  CB HB1
BOND CB HB2  SG O1 O1 H1
DOUBLE O  C
IMPR N -C CA HN  C CA +N O
CMAP -C  N  CA  C   N  CA  C  +N
DONOR HN N
DONOR H1 O1
ACCEPTOR O C
ACCEPTOR SG O1
IC -C   CA   *N   HN    1.3479 123.9300  180.0000 114.7700  0.9982
IC -C   N    CA   C     1.3479 123.9300  180.0000 105.8900  1.5202
IC N    CA   C    +N    1.4533 105.8900  180.0000 118.3000  1.3498
IC +N   CA   *C   O     1.3498 118.3000  180.0000 120.5900  1.2306
IC CA   C    +N   +CA   1.5202 118.3000  180.0000 124.5000  1.4548
IC N    C    *CA  CB    1.4830 111.2000  125.1000 116.1800  1.5584
IC N    C    *CA  HA    1.4830 111.2000 -118.3000 105.2000  1.0837
IC N    CA   CB   SG    1.5330 109.1000 -132.2000 114.0000  1.8280
IC SG   CA   *CB  HB1   1.8280 114.0000  118.2000 110.7000  1.1134
IC SG   CA   *CB  HB2   1.8280 114.0000 -122.4000 112.7000  1.1124
IC CA   CB   SG   O1    1.5320 114.0000  -49.8000  99.8000  1.6580
IC CB   SG   O1   H1    1.8280  99.8000  -75.7000 113.7000  0.9940
END
```

>>>Parameters included in CHARMM forcefield parameter file

```
BONDS
!
!V(bond) = Kb(b - b0)**2
!Kb: kcal/mole/A**2
!b0: A
!
!atom type Kb        b0
SO1  OH1   200.000     1.7090 !
SO1  CT2   198.000     1.8230 !

ANGLES
!
!V(angle) = Ktheta(Theta - Theta0)**2
!Ktheta: kcal/mole/rad**2
!Theta0: degrees
!
!atom types     Ktheta    Theta0
CT1  CT2  SO1    50.000   115.3000 !
SO1  OH1  H      50.000   110.1000 !
CT2  SO1  OH1    50.000    99.0000 !
SO1  CT2  HA2    46.100   107.0000 !

DIHEDRALS
```

```
!
!V(dihedral) = Kchi(1 + cos(n(chi) - delta))
!Kchi: kcal/mole
!n: multiplicity
!delta: degrees
!
!atom types             Kchi    n   delta
CT1  CT2  SO1  OH1      0.2000  3     0.00 !
NH1  CT1  CT2  SO1      0.2000  3     0.00 !
H    C    CT2  SO1      0.2000  3     0.00 !
C    CT2  SO1  OH1      0.2000  3     0.00 !
HA2  CT2  SO1  OH1      0.2000  3     0.00 !
H    OH1  SO1  CT2      1.1000  2     0.00 !
```

Supplementary Tables

Supplementary Table 1 | Cysteine conservation within the Src-family kinases

	185	238	245	277	400	483	487	496	498
**Src**	C	C	C	C	C	C	C	C	C
Yes	S	C	C	C	C	C	C	N	C
Fgr	S	C	C	C	C	C	C	E	T
Fyn	S	C	C	Q	C	C	C	I	C
Lck	S	C	C	Q	C	R	C	R	C
Hck	S	C	C	Q	C	R	C	M	C
Blk	S	C	C	Q	C	R	C	A	C
Lyn	S	C	C	Q	C	R	C	K	C
Frk	S	C	C	Q	Y	Q	C	L	C

Supplementary Table 2 | MS data of representative cysteine-containing peptides from Src modified by dimedone, BCN, or NBD, during oxidation by H_2O_2.

a) Src + 500 µM H_2O_2 + dimedone

Cysteine	Peptide Sequence	XCorr	Charge	MH+ (Da)	ΔM (Da)
Cys-185	GAYC*LSVSDFDNAK	3.26	+2	1626.82	-0.46
Cys-277	LGQGC*FGEVWMGTWNGTTR	4.56	+2	2239.28	0.63
Cys-498	MPC#PPEC#PESLHDLMC#QC*WRK	3.52	+3	2813.58	0.47

b) Src + 500 µM H_2O_2 + BCN

Cysteine	Peptide Sequence	XCorr	Charge	MH+ (Da)	ΔM (ppm)
Cys-185	GAYC†LSVSDFDNAK	2.65	+2	1655.76816	1.34
Cys-245	LTTVC†PTSKPQTQGLAK	3.86	+2	1940.05826	-2.5
Cys-277	LGQGC†FGEVWMGTWNGTTR	3.97	+2	2266.03911	-4.66

c) Src + 200 µM H_2O_2 + NBD-Cl

Cysteine	Peptide Sequence	XCorr	Charge	MH+ (Da)	ΔM (ppm)
Cys-277	LGQGC@FGEVWMGTWNGTTR	3.12	+2	2280.012	-470.4358
Cys-498	MPC#PPEC#PESLHDLMC#QC@WRK	4.2	+3	2724.031	-362.306

Peptides listed were identified in a dataset limited to <1% FP
*-denotes dimedone tagged cysteine residue (+138.068 Da).
†-denotes BCN-S(O)-tagged cysteine residue (+166.099 Da).
@-denotes NBD-S(O)-tagged cysteine residue (+178.997 Da).
#-denotes carbamidomethylated reduced cysteine residue (+57.0215 Da).

Supplementary Table 3 | Summary of all-atom MD simulations

System	Box size ($Å^3$)	No. of atoms	No. of water molecules	Simulation lengths (μs)
Autoinhibited Src (wild-type)	94×94×94	85439	26037	5.0, 2.2
C185-SOH	85×89×82	58370	17030	5.1, 5.1
C277-SOH	85×89×82	58370	17030	5.5, 5.2
C498-SOH	87×90×83	60699	17805	5.0, 5.0
**Metadynamics simulations**				Simulation lengths (ns)
Wild-type (C185-SH)	94×94×94	85439	26037	120
Wild-type (C277-SH)	94×94×94	85439	26037	120
Wild-type (C498-SH)	94×94×94	85439	26037	120
C185-SOH	85×89×82	58370	17030	120
C277-SOH	85×89×82	58370	17030	120
C498-SOH	87×90×83	60699	17805	120

Supplementary Figures

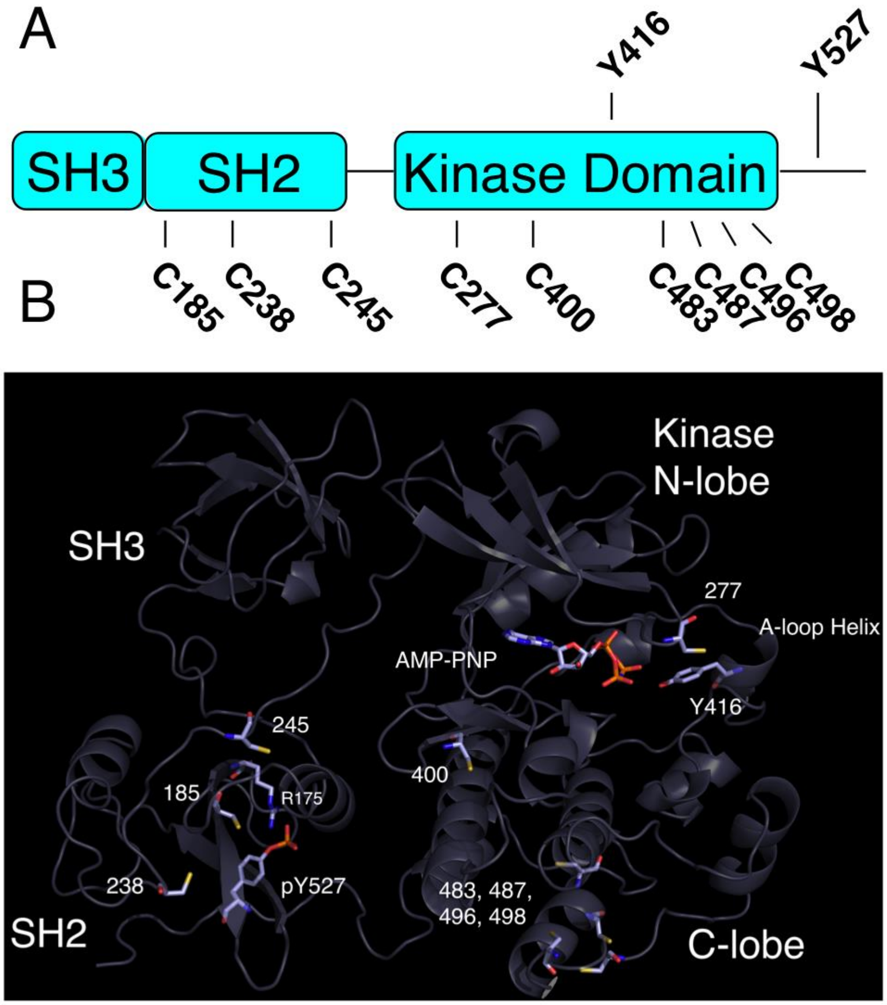

Supplementary Figure 1 | Localization of 9 cysteine residues within Src. The Src kinase contains three cysteine residues within the SH2 domain and six within the kinase domain. A) Distribution of cysteine residues within the Src sequence. B) The autoinhibited structure of Src (PDB ID 2SRC) with cysteine residues, Tyr-416, pTyr-527, and ATP binding site.

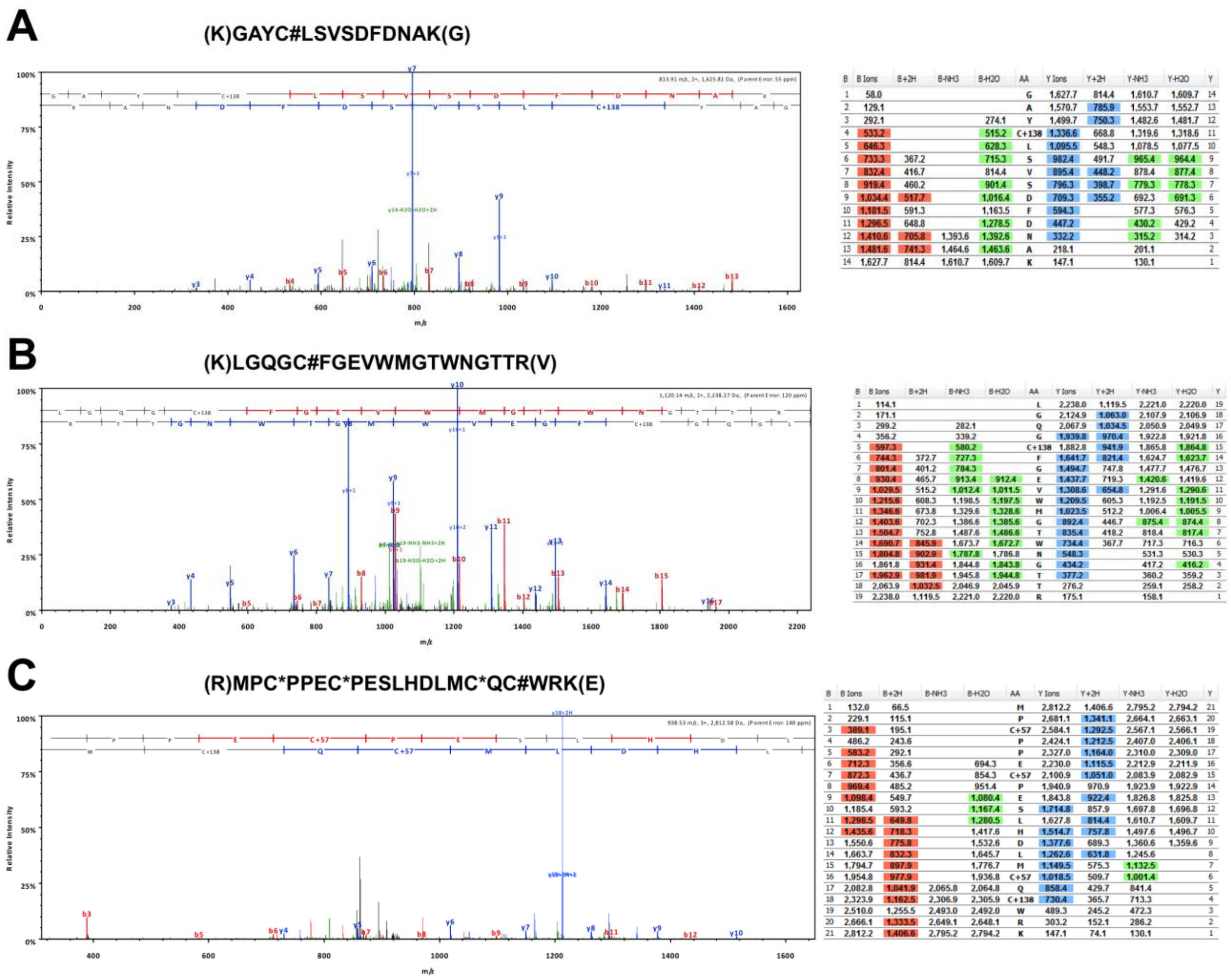

Supplementary Figure 2 | Identification of H_2O_2-oxidizable Cys residues in Src by dimedone trapping. Recombinant Src (~0.6 µg/µL) was reacted with 0.5 mM H_2O_2 in the presence of ~0.03 mM DTT and 1.0 mM dimedone and trypsin digested for LC-MS/MS analysis. Spectra of peptides and ions observed for: A) Cys-185 GAYC#LSVSDFDNAK, B) Cys-277 LGQGC#FGEVWMGTWNGTTR, C) Cys-498 MPCPPECPESLHDLMCQC#WRK where #- indicates a dimedone tagged cysteine. Peptides were identified in a dataset limited to <1% FP and the corresponding MSMS spectra visualized with Scaffold 4.05.

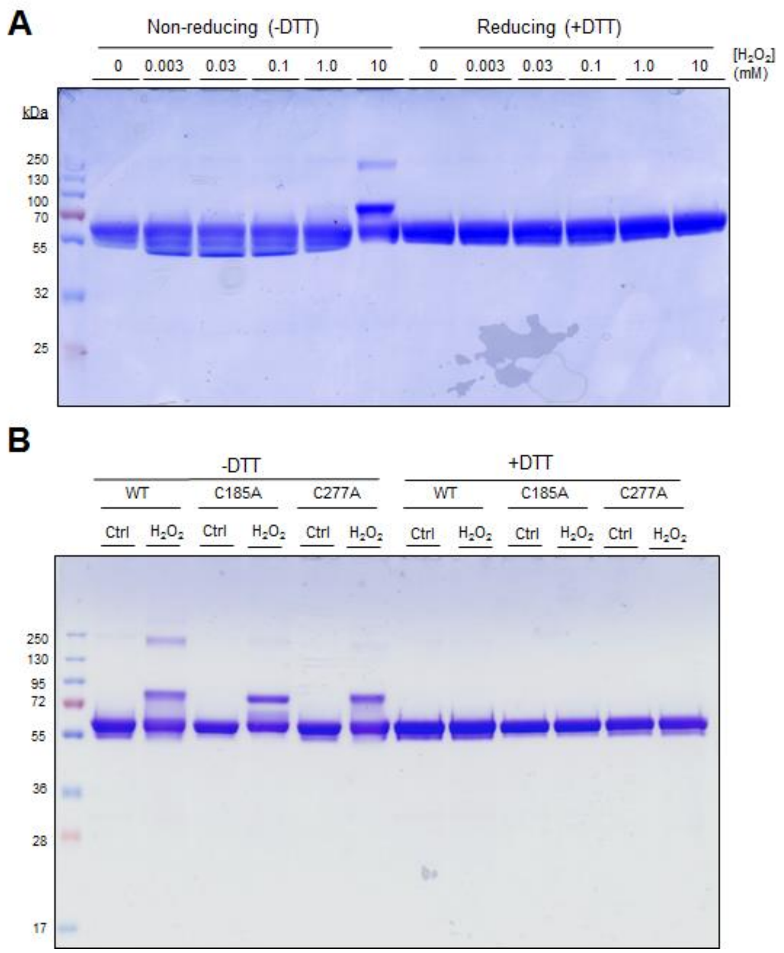

Supplementary Figure 3 | H_2O_2 induces disulfide-mediated crosslinking in recombinant Src. A) Src (~10 µg) was incubated with indicated concentrations of H_2O_2, and analyzed by SDS-PAGE and Coomassie stained. DTT-reducible shifts in several high-molecular weight bands were detected in response to increasing concentrations of H_2O_2, suggesting the formation of intra- or intermolecular disulfides. B) Formation of inter- or intramolecular disulfides in WT or C185A or C227A variants of Src upon reaction with 20 mM H_2O_2.

A

(K)GAYC#LSVSDFDNAK(G)

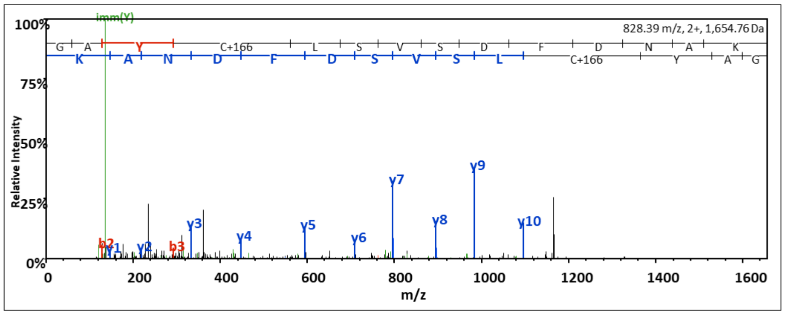

B	B Ions	B+2H	B-NH3	B-H2O	AA	Y Ions	Y+2H	Y-NH3	Y-H2O	Y
1	58.0				G	1,655.7	828.3	1,638.6	1,637.7	14
2	129.1				A	1,598.6	799.8	1,581.6	1,580.6	13
3	292.1			274.1	Y	1,527.6	764.3	1,510.6	1,509.6	12
4	561.1			543.1	C+166	1,364.5	682.8	1,347.5	1,346.5	11
5	674.2			656.2	L	1,095.5	548.3	1,078.5	1,077.5	10
6	761.3	381.1		743.2	S	982.4	491.7	965.4	964.4	9
7	860.3	430.7		842.3	V	895.4	448.2	878.4	877.4	8
8	947.4	474.2		929.3	S	796.3	398.7	779.3	778.3	7
9	1,062.4	531.7		1,044.4	D	709.3	355.2	692.3	691.3	6
10	1,209.5	605.2		1,191.4	F	594.3		577.3	576.3	5
11	1,324.5	662.7		1,306.5	D	447.2		430.2	429.2	4
12	1,438.5	719.8	1,421.5	1,420.5	N	332.2		315.2	314.2	3
13	1,509.6	755.3	1,492.5	1,491.5	A	218.1		201.1		2
14	1,655.7	828.3	1,638.6	1,637.7	K	147.1		130.1		1

B

(R)LTTVC#PTSKPQTQGLAK(D)

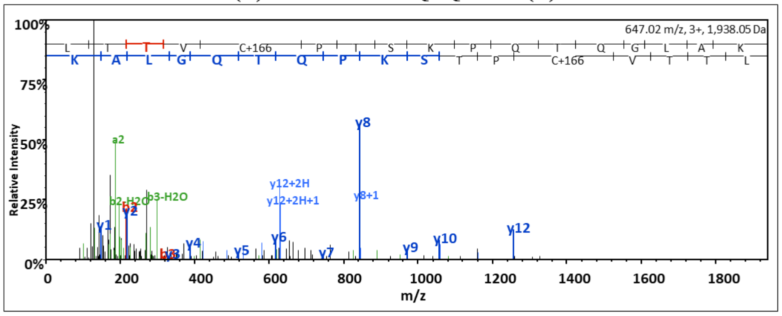

B	B Ions	B+2H	B-NH3	B-H2O	AA	Y Ions	Y+2H	Y-NH3	Y-H2O	Y
1	114.1	57.5			L	1,939.0	970.0	1,921.9	1,920.9	17
2	215.1	108.1		197.1	T	1,825.9	913.4	1,808.8	1,807.9	16
3	316.2	158.6		298.2	T	1,724.8	862.9	1,707.8	1,706.8	15
4	415.3	208.1		397.2	V	1,623.8	812.4	1,606.8	1,605.8	14
5	684.3	342.6		666.3	C+166	1,524.7	762.9	1,507.7	1,506.7	13
6	781.3	391.2		763.3	P	1,255.7	628.4	1,238.7	1,237.7	12
7	882.4	441.7		864.4	T	1,158.6	579.8	1,141.6	1,140.6	11
8	969.4	485.2		951.4	S	1,057.6	529.3	1,040.6	1,039.6	10
9	1,097.5	549.3	1,080.5	1,079.5	K	970.6	485.8	953.5	952.6	9
10	1,194.5	597.8	1,177.5	1,176.5	P	842.5	421.7	825.4	824.5	8
11	1,322.6	661.8	1,305.6	1,304.6	Q	745.4	373.2	728.4	727.4	7
12	1,423.7	712.3	1,406.6	1,405.6	T	617.4	309.2	600.3	599.4	6
13	1,551.7	776.4	1,534.7	1,533.7	Q	516.3	258.7	499.3		5
14	1,608.7	804.9	1,591.7	1,590.7	G	388.3	194.6	371.2		4
15	1,721.8	861.4	1,704.8	1,703.8	L	331.2	166.1	314.2		3
16	1,792.9	896.9	1,775.8	1,774.8	A	218.1	109.6	201.1		2
17	1,939.0	970.0	1,921.9	1,920.9	K	147.1	74.1	130.1		1

C

(K)LGQGC#FGEVWMGTWNGTTR(V)

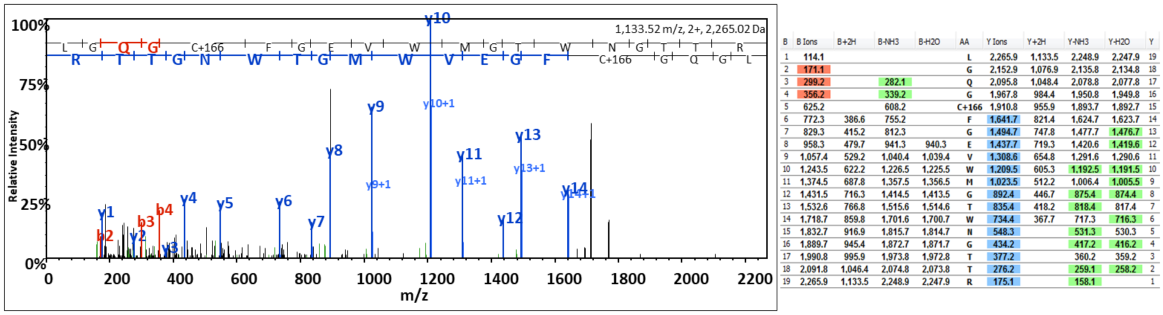

B	B Ions	B+2H	B-NH3	B-H2O	AA	Y Ions	Y+2H	Y-NH3	Y-H2O	Y
1	114.1				L	2,265.9	1,133.5	2,248.9	2,247.9	19
2	171.1				G	2,152.9	1,076.9	2,135.8	2,134.8	18
3	299.2		282.1		Q	2,095.8	1,048.4	2,078.8	2,077.8	17
4	356.2		339.2		G	1,967.8	984.4	1,950.8	1,949.8	16
5	625.2		608.2		C+166	1,910.8	955.9	1,893.7	1,892.7	15
6	772.3	386.6	755.2		F	1,641.7	821.4	1,624.7	1,623.7	14
7	829.3	415.2	812.3		G	1,494.7	747.8	1,477.7	1,476.7	13
8	958.3	479.7	941.3	940.3	E	1,437.7	719.3	1,420.6	1,419.6	12
9	1,057.4	529.2	1,040.4	1,039.4	V	1,308.6	654.8	1,291.6	1,290.6	11
10	1,243.5	622.2	1,226.5	1,225.5	W	1,209.5	605.3	1,192.5	1,191.5	10
11	1,374.5	687.8	1,357.5	1,356.5	M	1,023.5	512.2	1,006.4	1,005.5	9
12	1,431.5	716.3	1,414.5	1,413.5	G	892.4	446.7	875.4	874.4	8
13	1,532.6	766.8	1,515.6	1,514.6	T	835.4	418.2	818.4	817.4	7
14	1,718.7	859.8	1,701.6	1,700.7	W	734.4	367.7	717.3	716.3	6
15	1,832.7	916.9	1,815.7	1,814.7	N	548.3		531.3	530.3	5
16	1,889.7	945.4	1,872.7	1,871.7	G	434.2		417.2	416.2	4
17	1,990.8	995.9	1,973.8	1,972.8	T	377.2		360.2	359.2	3
18	2,091.8	1,046.4	2,074.8	2,073.8	T	276.2		259.1	258.2	2
19	2,265.9	1,133.5	2,248.9	2,247.9	R	175.1		158.1		1

Supplementary Figure 4 | Identification of H_2O_2-oxidizable Cys residues in Src by trapping with BCN. Recombinant Src (~0.4 µg/µL) was reacted with 0.5 mM H_2O_2 in the presence of ~0.25 mM DTT and 100 µM BCN and trypsin digested for LC-MS/MS analysis. Spectra of peptides and ions observed for: A) Cys-185 GAYC#LSVSDFDNAK, B) Cys-245 LTTVC#PTSKPQTQGLAK C) Cys-277 LGQGC#FGEVWMGTWNGTTR, where # indicates a BCN tagged cysteine. Peptides were identified in a dataset limited to <1% FP and the corresponding MSMS spectra visualized with Scaffold 4.05.

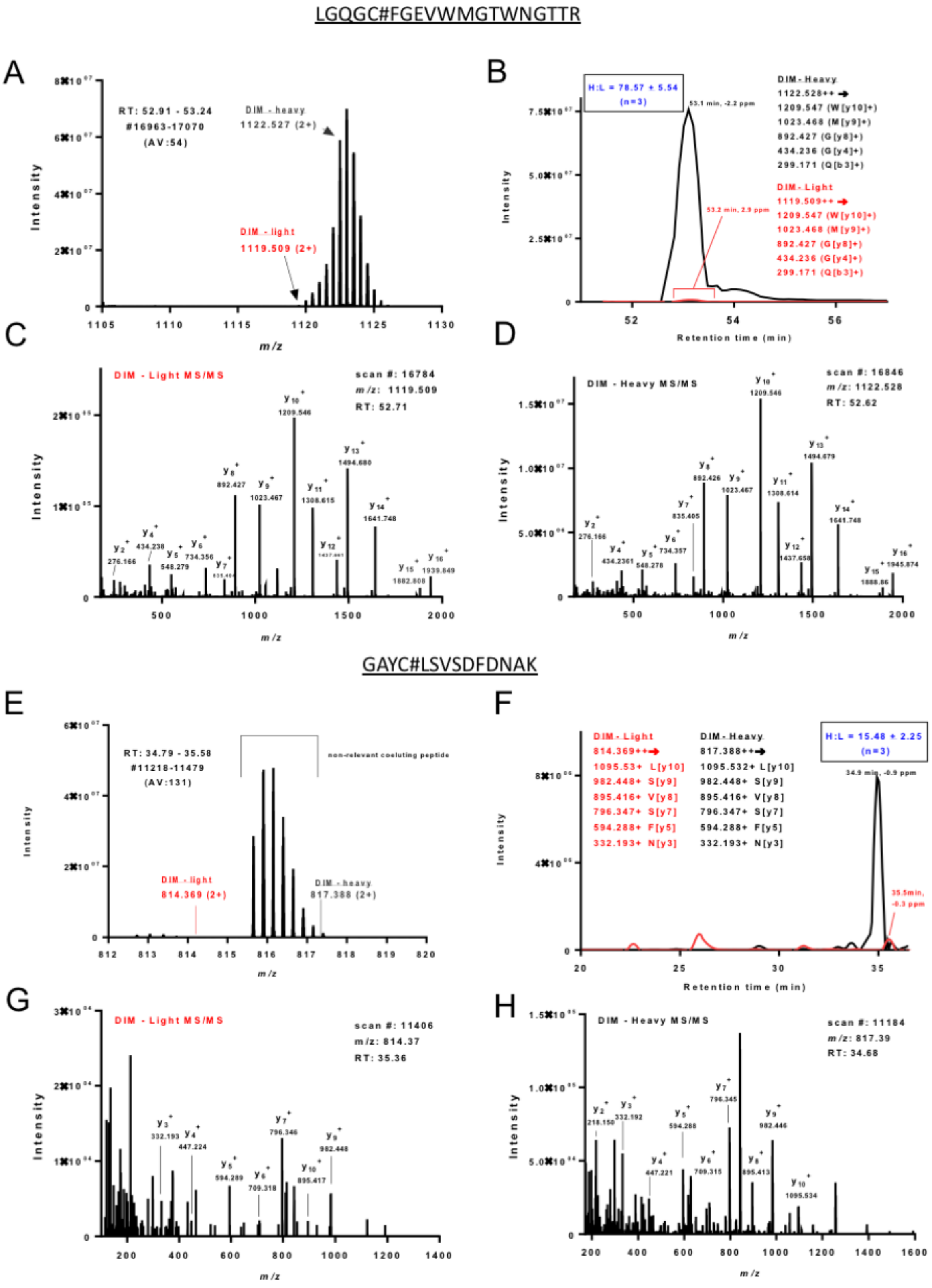
LGQGC#FGEVWMGTWNGTTR
A
RT: 52.91 - 53.24
#16963-17070
(AV:54)
DIM - heavy
1122.527 (2+)
DIM - light
1119.509 (2+)
Intensity
m/z
B
H:L = 78.57 ± 5.54
(n=3)
53.1 min, -2.2 ppm
53.2 min, 2.9 ppm
DIM - Heavy
1122.528++
1209.547 (W[y10]+)
1023.468 (M[y9]+)
892.427 (G[y8]+)
434.236 (G[y4]+)
299.171 (Q[b3]+)
DIM - Light
1119.509++
1209.547 (W[y10]+)
1023.468 (M[y9]+)
892.427 (G[y8]+)
434.236 (G[y4]+)
299.171 (Q[b3]+)
Retention time (min)
C
DIM - Light MS/MS
scan #: 16784
m/z: 1119.509
RT: 52.71
D
DIM - Heavy MS/MS
scan #: 16846
m/z: 1122.528
RT: 52.62
GAYC#LSVSDFDNAK
E
RT: 34.79 - 35.58
#11218-11479
(AV:131)
non-relevant coeluting peptide
DIM - light
814.369 (2+)
DIM - heavy
817.388 (2+)
F
DIM - Light
814.369++
1095.53+ L[y10]
982.448+ S[y9]
895.416+ V[y8]
796.347+ S[y7]
594.288+ F[y5]
332.193+ N[y3]
DIM - Heavy
817.388++
1095.532+ L[y10]
982.448+ S[y9]
895.416+ V[y8]
796.347+ S[y7]
594.288+ F[y5]
332.193+ N[y3]
H:L = 15.48 ± 2.25
(n=3)
34.9 min, -0.9 ppm
35.5min, -0.3 ppm
G
DIM - Light MS/MS
scan #: 11406
m/z: 814.37
RT: 35.36
H
DIM - Heavy MS/MS
scan #: 11184
m/z: 817.39
RT: 34.68

Supplementary Figure 5 | Quantitative analysis of Src sulfenylation using LC-MS/MS. A) MS1 spectrum of the dimedone-labeled (DIM light) and deuterated dimedone (DIM-d6 heavy) labeled LGQGCFGEVWMGTWNGTTR (containing Cys-277). Corresponding precursors (at m/z 1119.53 and m/z 1122.53, respectively) are indicated. PRM was simultaneously carried out with MS-SIM in the same workflow, as described in the Materials and Methods. The transitions for quantification were selected using the Skyline software. B) Corresponding extracted ion chromatograms of the isotopologues eluting at around the same retention time (with deuterated version eluting slightly earlier). The peaks of elution are indicated by arrows. H/L ratio was calculated from H/L ratios of the 5 transitions from 3 experimental replicates. C,D) MS/MS spectra of LGQGC(dim)FGEVWMGTWNGTTR and LGQGC(dim-d6)FGEVWMGTWNGTTR acquired during peak elution of the target, and annotated according to the Proteome Discoverer search results. E) MS1 spectrum of the dimedone-labeled (DIM light) and deuterated dimedone (DIM-d6 heavy) labeled GAYC(dim/dim-d6)LSVSDFDNAK peptide (which contains Cys-185). Due to low abundance, no corresponding precursors (at m/z 814.369 and m/z 817.338, respectively) were observable in the MS-SIM scans (*top left*), and their quantification were carried out using parallel reaction monitoring (PRM). F) Extracted ion chromatograms of the PRM transitions of the isotopologues eluting at around the same retention time (with deuterated version eluting slightly earlier). The peaks of elution are indicated by arrows. The heavy/light (H/L) ratio was calculated from H/L ratios of the six transitions from 3 experimental replicates. G,H) MS/MS spectra of GAYC(dim)LSVSDFDNAK and GAYC(dim-d6)LSVSDFDNAK acquired during peak elution of the target and annotated according to the Proteome Discoverer search results.

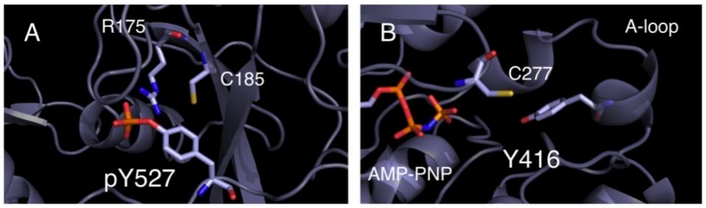

Supplementary Figure 6 | Autoinhibited Src (PDB: 2SRC) structure zoom in of A) Cys-185 near pTyr-527 and Arg-175 and B) Cys-277 near the ATP binding site and A-loop.

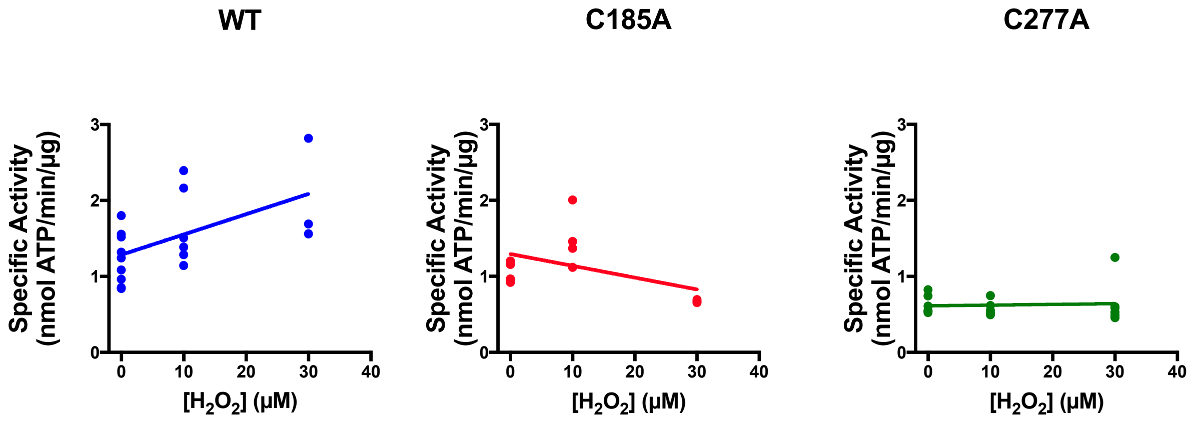

Supplementary Figure 7 | Correlation of Src specific activity with H_2O_2 concentration for WT and mutant proteins. Plots of specific activity versus [H_2O_2] and linear regression analysis of WT, C185A, and C277A Src recombinant protein (based on data presented in Figure 2B). Least squared fits to straight lines and correlation analyses show WT Src specific activity is linearly dependent on [H_2O_2] (slope = +0.027, Pearson r = +0.98) while C185A (slope = -0.016, Pearson r = -0.63) and C277A (slope = +0.00095, Pearson r = +0.45) are not impacted by H_2O_2.

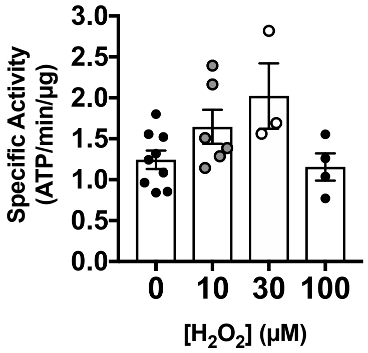

Supplementary Figure 8 | H_2O_2-dependent wild-type Src tyrosine kinase activity at 100 µM H_2O_2. Effect of H_2O_2 on tyrosine kinase activity of WT recombinant Src and measured with ADP Glo kinase activity kit (same as in Fig. 3B at 0, 10 and 30 µM). Data are expressed as means ± s.e.m. of at least 3 replicates from 3 independent experiments.

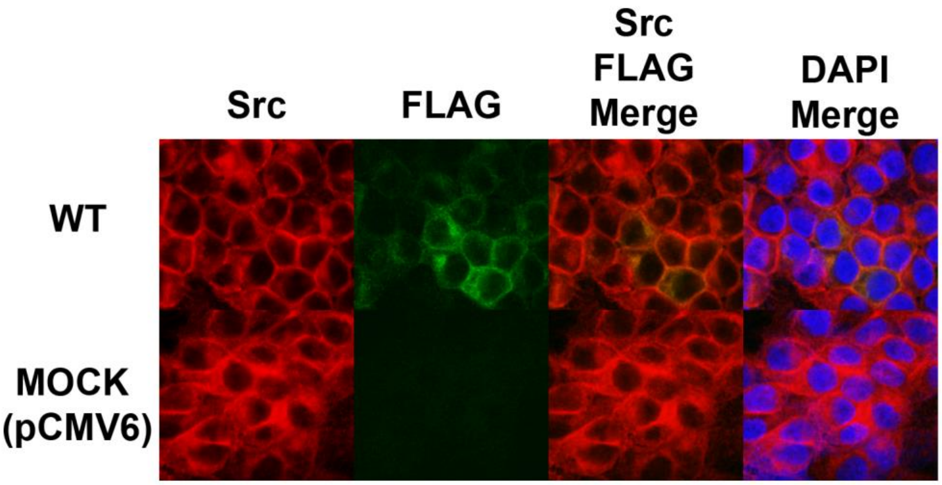

Supplementary Figure 9 | FLAG-Src co-localizes with endogenous Src in transfected stable H292 cell lines. Immunofluorescent analysis of WT FLAG-Src or MOCK transfection (pCMV6 empty vector) transfected H292 cells. Cells were stained with both α-Src (red) and α-FLAG (green) primary antibodies and corresponding fluorescent secondary antibodies. Nuclei were counterstained with DAPI (blue).

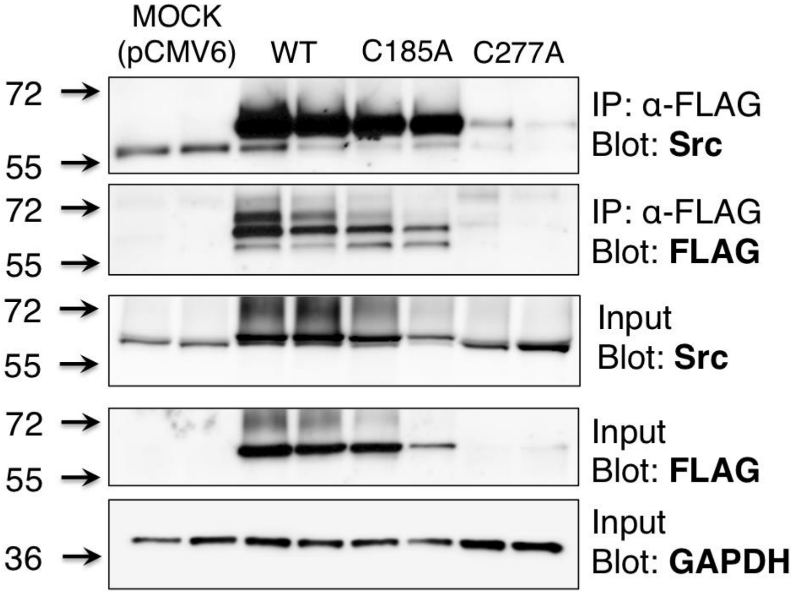

Supplementary Figure 10 | Western blot analysis of stable H292 cell lines expressing WT, C185A, and C277A Src compared to MOCK transfected cells. Blots indicate that FLAG-tagged Src protein at a molecular weight of ~65 kDa and non-specific binding at ~60 kDa after FLAG immunoprecipitation with M2 anti-FLAG magnetic beads.

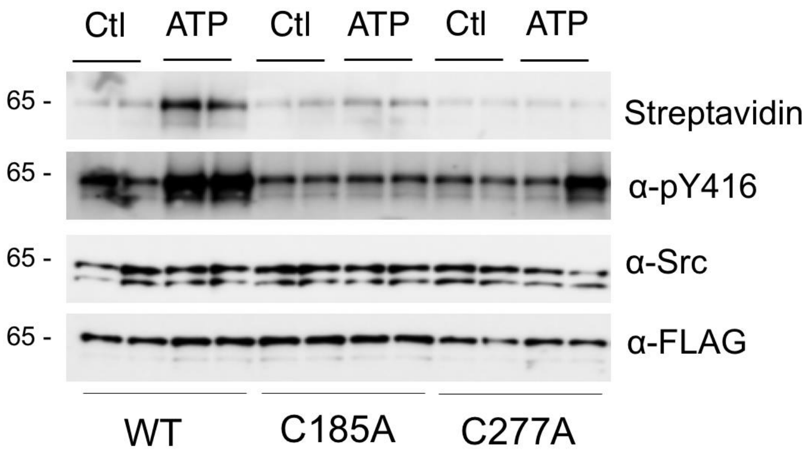

Supplementary Figure 11 | ATP stimulation of H292 cells causes Src sulfenylation at Cys-185 and Cys-277 which promote pTyr-416 phosphorylation. A) NCI-H292 cells stably transfected with c-terminal FLAG-tagged WT, C185A, and C277A Src (Src-FLAG) were preloaded with DYn-2 and stimulated with 100 µM ATP for 10 min. Derivatized cell lysates or α-FLAG-purified were analyzed by Western blot with indicated antibodies. Representative Western blots of 2 separate experiments are shown.

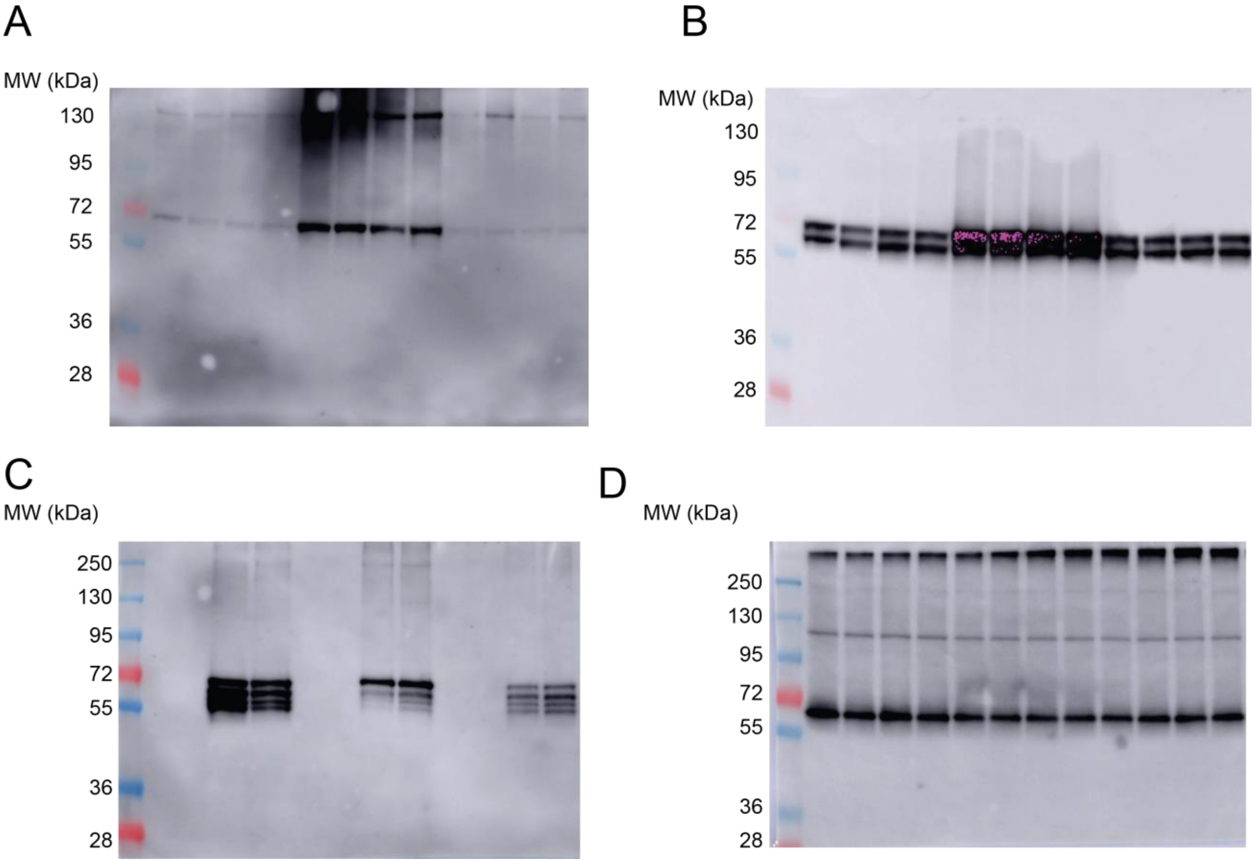

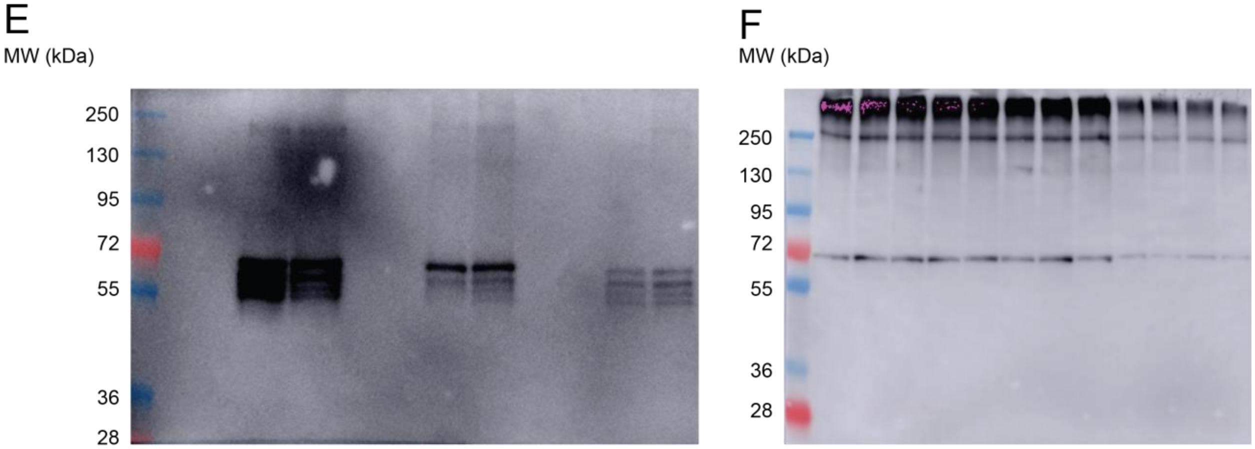

Supplementary Figure 12 | Full membrane images for corresponding Western blots presented in Fig. 3 with molecular weight markers. H292 cells were transiently transfected with c-terminal FLAG-tagged WT, C185A, and C277A Src (Src-FLAG), and preloaded with DYn-2 and stimulated with 100 μM ATP for 10 min. Complete Western blot visualized with A) α-Src and B) α-FLAG from cell lysates and C) α-streptavidin, D) α-Src, E) α-pY416, and F) α-FLAG from α-FLAG-purified lysates.

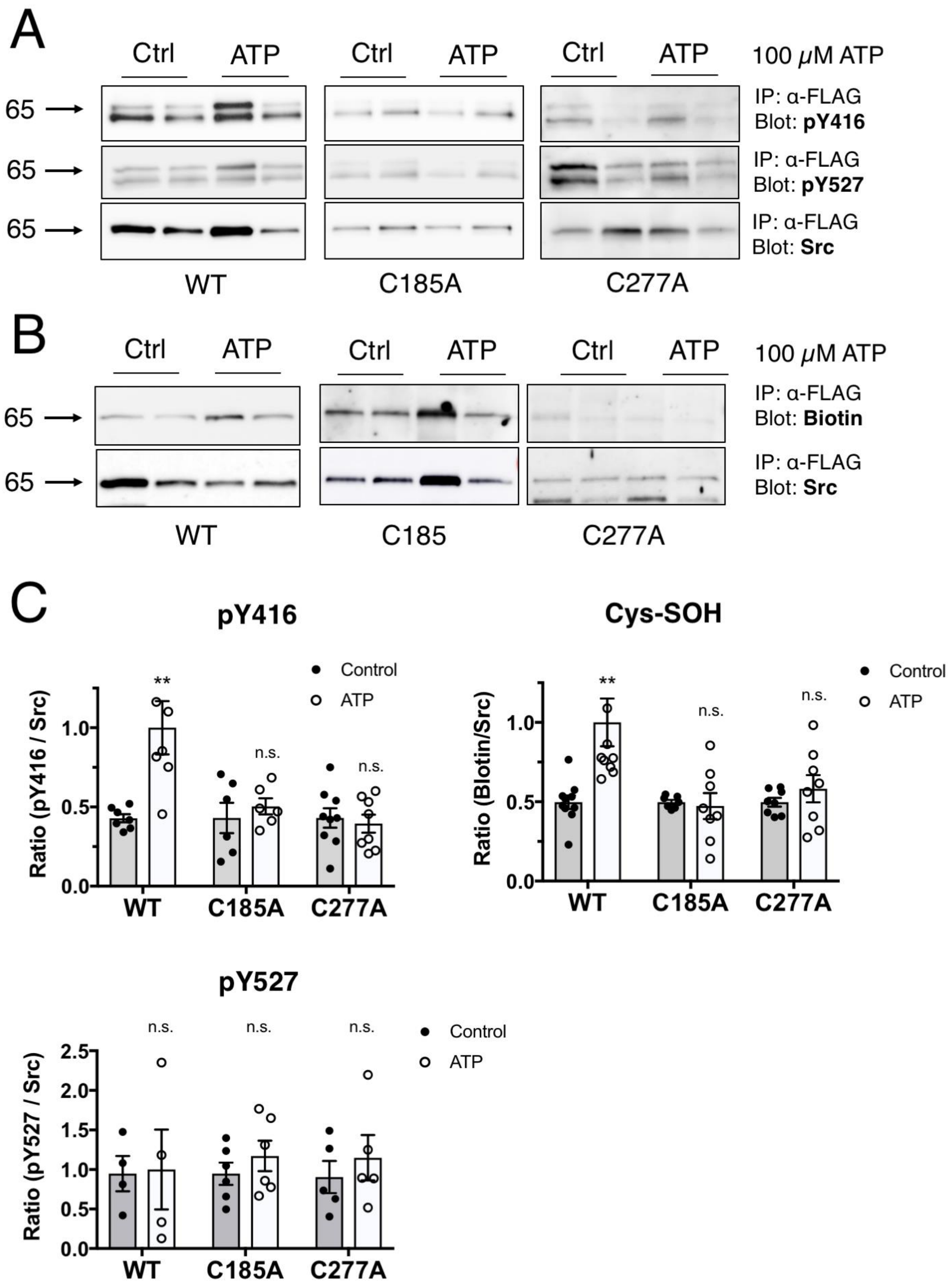

Supplementary Figure 13 | ATP-dependent stimulation of H292 cells requires Src Cys-185 and Cys-277 for pTyr-416 phosphorylation in association with sulfenylation of Cys-185 and Cys-277. A) NCI-H292 cells stably expressing c-terminal FLAG-tagged WT, C185A, and C277A Src (Src-FLAG) were stimulated with 100 µM ATP for 10 min and changes in Src phosphorylation at Tyr-416 and Tyr-527 were analyzed by Western blot after FLAG-tag protein purification. B) Cells were stimulated as in A), but lysed in the presence of DCP-bio1 to

conjugate with Cys-SOH species followed by purification of FLAG-tag proteins and analysis Cys-SOH amounts by blotting for biotin conjugation to WT, C185A, and C277A Src-FLAG. C) Normalized ratios of phospho-to-total Src or biotinylation-to-total Src. Western blot experiments are representative of at least 3 independent experiments. Data represent mean ± s.e.m of phosphorylation changes of at least 4 replicates, normalized ratio of phosphorylation/total WT ATP-treated of 1.0. Data was analyzed with student *t* test comparing control to ATP treated cells ** $p < 0.01$.

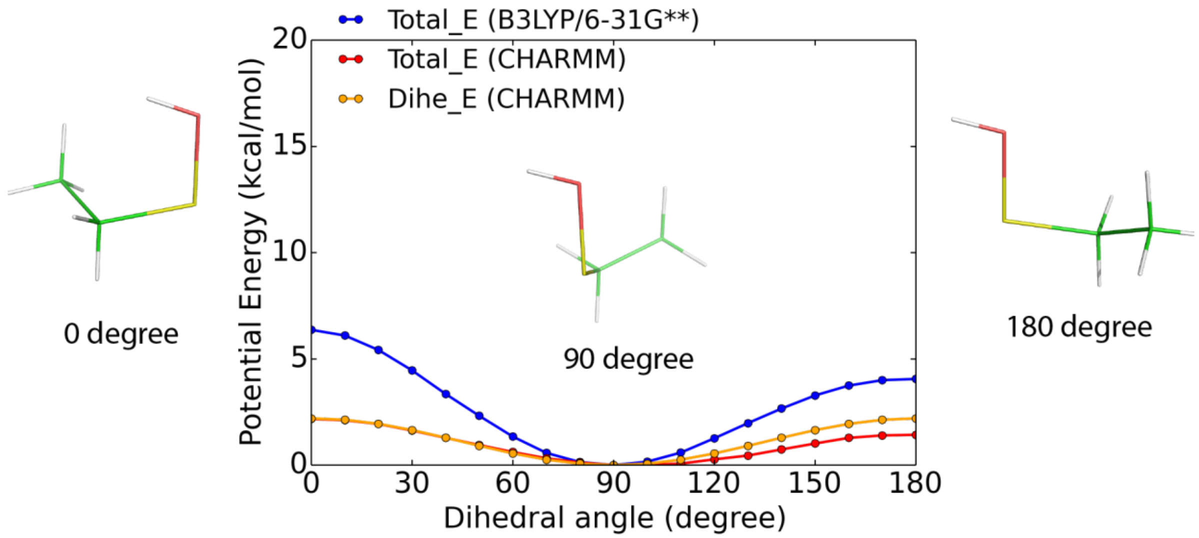

Supplementary Figure 14 | Dihedral profiles of –C-S-O-H from DFT calculations using B3LYP/6-31G** (blue), total potential (red) and dihedral term energy (orange) with our parameters implemented in CHARMM force field. A model molecule, CH3-CH2-SOH, is used to compare our parameters for -SOH with DFT calculations geometrically and energetically in dihedral scanning. Three optimized conformation with dihedral angle as 0, 90, 180 degrees are displayed.

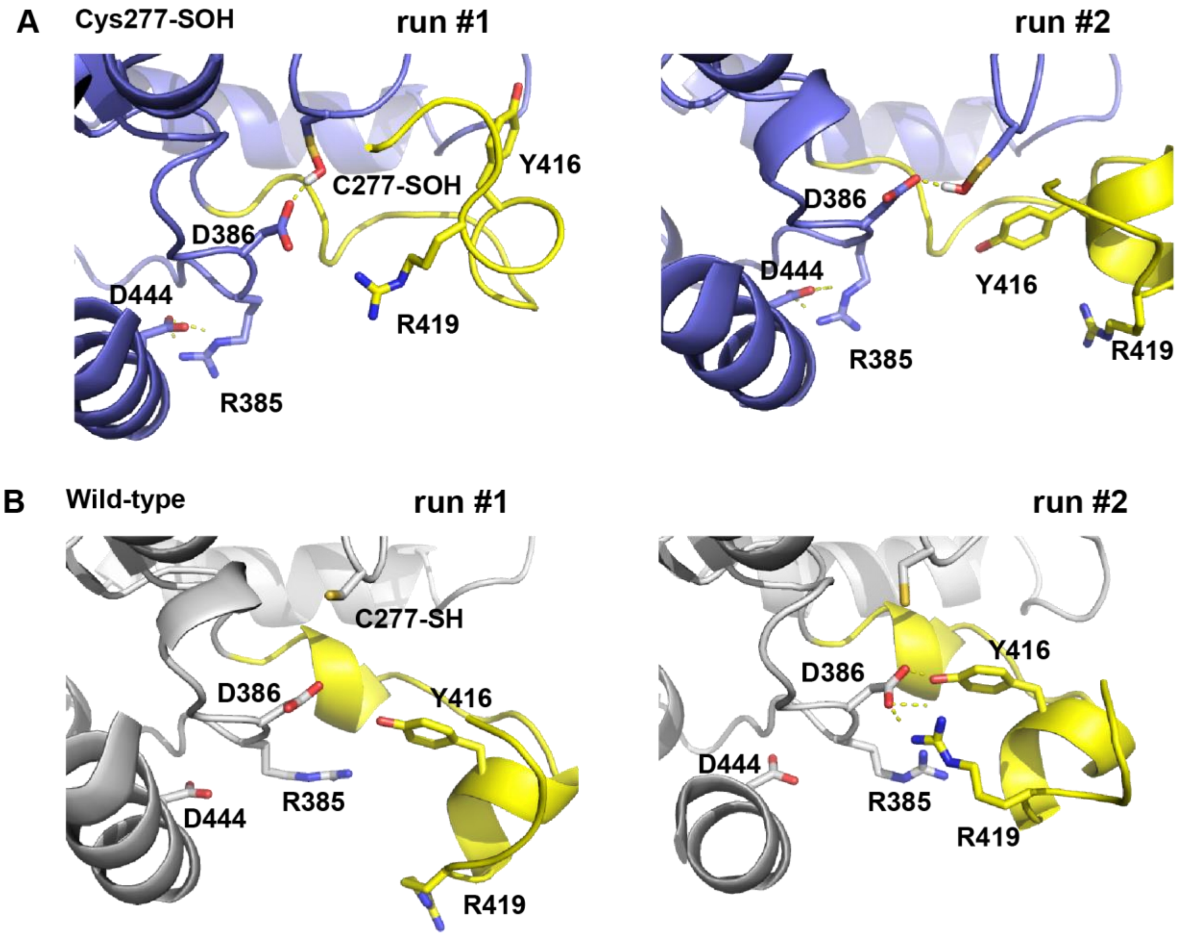

Supplementary Figure 15 | (A) Final structures of Cys-277-SOH from two MD replicas labeled as #1 and #2. (B) Final structures of wild-type group from two MD replicas labeled as #1 and #2. The cysteine oxidation to Cys-277-SOH induced the helicity loss at residues 406 to 423 (yellow). Key residues that interact with the A-loop region are labeled. Cys-277-SOH is likely to compete with Tyr-416 to interact with Asp-386, which weaken the interactions of Tyr-416 with the nearby residues. Also, Arg-385 was found to pair with Asp-444 by hydrogen bonding when Cys-277-SOH interacts with Asp-386, which induces a displacement of the loop (res. 383-389) that contains Asp-386 and Arg-385 (see Figure S13B).

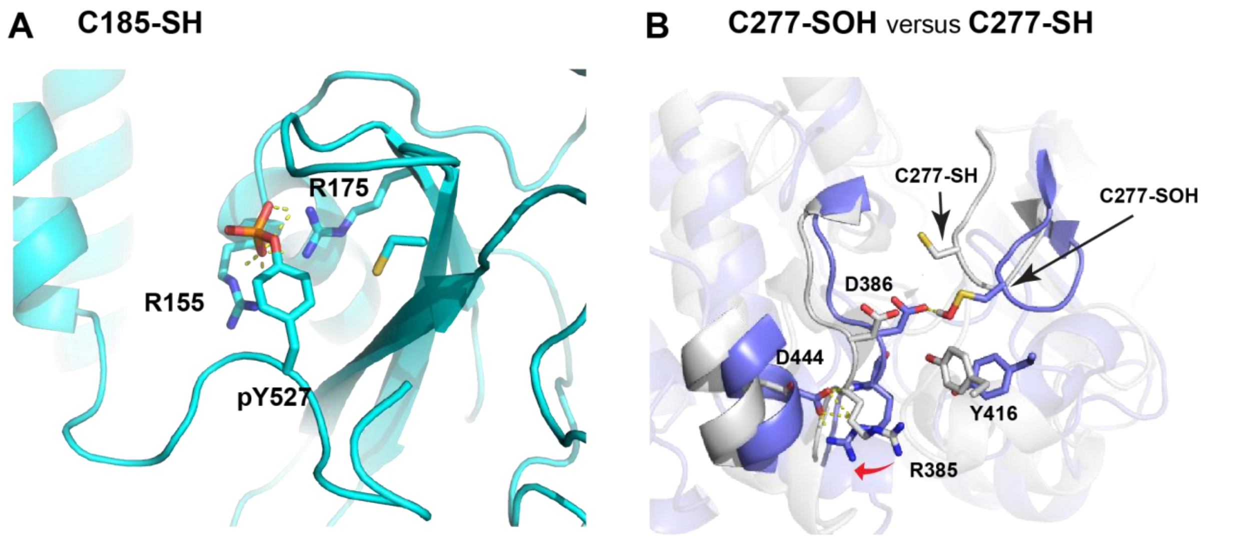

Supplementary Figure 16 | (A) A final snapshot of Cys-185-SH system. (B) Displacement of loop res. 383-389 associated with Cys-277-SOH - Asp-386 pair in Cys-277-SOH system (purple blue) superposed with the wild-type group (grey).

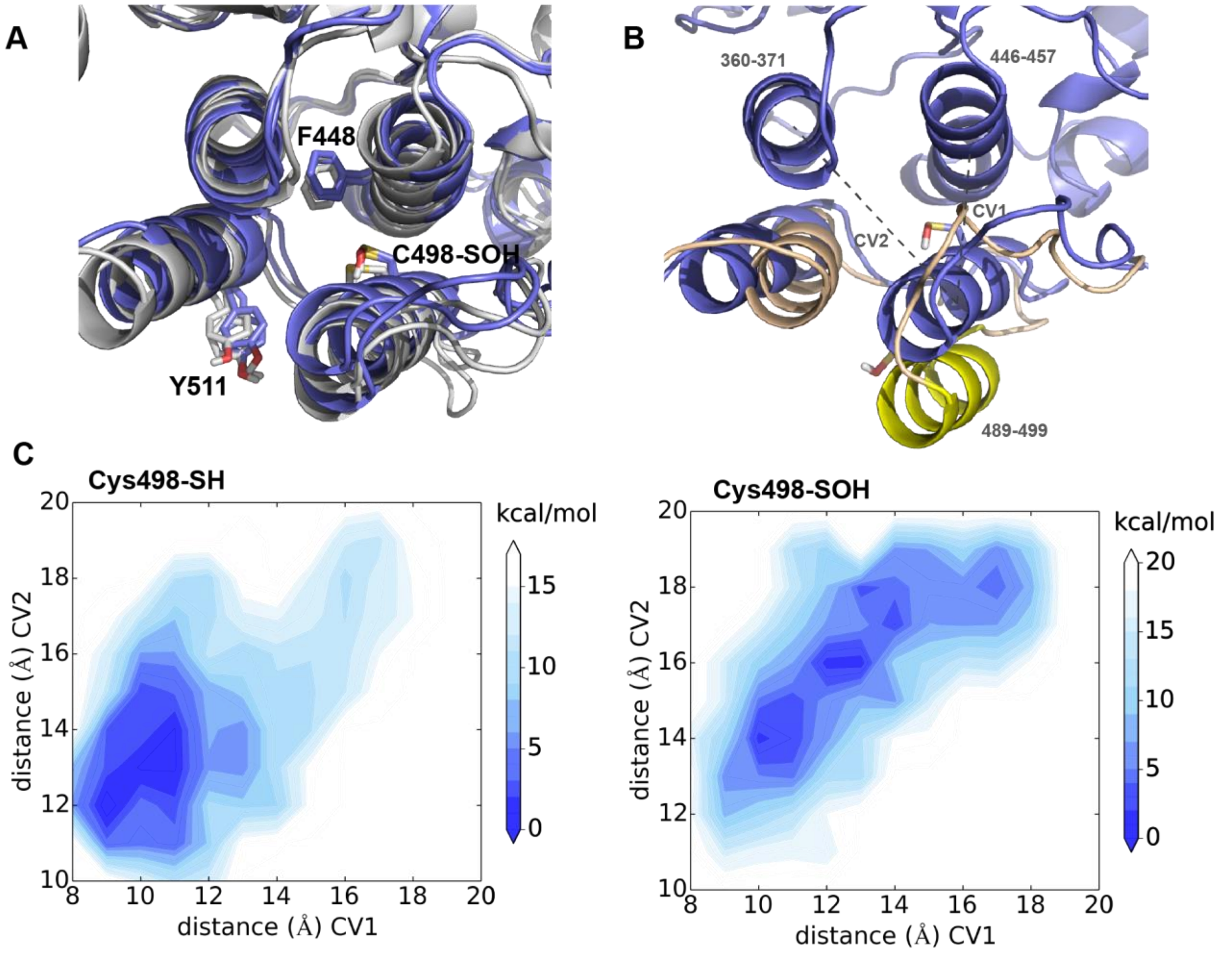

Supplementary Figure 17 | (A) Structural superpositions of Cys-498-SOH system (light blue) with wild-type groups (grey). Final snapshots from two replicas are presented for each system. (B) Two collective variables, center-of-mass distances of helical res. 489-499 and helical res. 446-457 and res. 360-371 respectively, are used to characterize the dissociation of helical res. 489-499 from the helices bundle in metadynamics simulations. The displacement of res. 489-499 from metadynamics simulation is in yellow cartoon with reference to a final snapshot from MD simulation. (C) Free-energy landscapes of wild-type group (left panel) and Cys-498-SOH system (right panel). Comparable energy basins for the Cys-498-SH and Cys-498-SOH systems suggest that the oxidation of Cys-498 has a minor to absent impact on the dissociation of the C-lobe. Color Scale: Cys498-SOH white = 16.0 kcal/mol, blue = 0.0 kcal/mol (left) and Cys498-SH white = 20.0 kcal/mol, blue = 0.0 kcal/mol.

Supplementary References

1. Seeliger MA*, et al.* High yield bacterial expression of active c-Abl and c-Src tyrosine kinases. *Protein Science* **14**, 3135-3139 (2005).

2. Conte ML, Carroll KS. The redox biochemistry of protein sulfenylation and sulfinylation. *Journal of Biological Chemistry* **288**, 26480-26488 (2013).

3. Paulsen CE, Carroll KS. Cysteine-Mediated Redox Signaling: Chemistry, Biology, and Tools for Discovery. *Chemical Reviews* **113**, 4633-4679 (2013).

4. Käll L, Canterbury JD, Weston J, Noble WS, MacCoss MJ. Semi-supervised learning for peptide identification from shotgun proteomics datasets. *Nature Methods* **4**, 923 (2007).

5. Xu W, Doshi A, Lei M, Eck MJ, Harrison SC. Crystal structures of c-Src reveal features of its autoinhibitory mechanism. *Mol Cell* **3**, 629-638 (1999).

6. Jo S, Kim T, Iyer VG, Im W. CHARMM-GUI: a web-based graphical user interface for CHARMM. *J Comput Chem* **29**, 1859-1865 (2008).

7. Vanommeslaeghe K*, et al.* CHARMM general force field: A force field for drug ‐ like molecules compatible with the CHARMM all ‐ atom additive biological force fields. *Journal of computational chemistry* **31**, 671-690 (2010).

8. Best RB*, et al.* Optimization of the Additive CHARMM All-Atom Protein Force Field Targeting Improved Sampling of the Backbone ϕ, ψ and Side-Chain χ1 and χ2 Dihedral Angles. *J Chem Theory Comput* **8**, 3257-3273 (2012).

9. Phillips JC*, et al.* Scalable molecular dynamics with NAMD. *Journal of computational chemistry* **26**, 1781-1802 (2005).

10. Shaw DE*, et al.* Millisecond-scale molecular dynamics simulations on Anton. In: *High performance computing networking, storage and analysis, proceedings of the conference on* (ed^(eds). IEEE (2009).

11. Clark AJ*, et al.* Prediction of Protein–Ligand Binding Poses via a Combination of Induced Fit Docking and Metadynamics Simulations. *Journal of Chemical Theory and Computation* **12**, 2990-2998 (2016).

12. Humphrey W, Dalke A, Schulten K. VMD: visual molecular dynamics. *J Mol Graph Model* **14**, 33-38 (1996).

13. Becke AD. Density ‐ functional thermochemistry. III. The role of exact exchange. *The Journal of chemical physics* **98**, 5648-5652 (1993).

14. Bochevarov AD*, et al.* Jaguar: A high - performance quantum chemistry software program with strengths in life and materials sciences. *International Journal of Quantum Chemistry* **113**, 2110-2142 (2013).

15. Wildman J, Repiščák P, Paterson MJ, Galbraith I. General Force-Field Parametrization Scheme for Molecular Dynamics Simulations of Conjugated Materials in Solution. *Journal of Chemical Theory and Computation* **12**, 3813-3824 (2016).
