# Supplementary material for: Direct cysteine sulfenylation drives activation of the Src kinase
Source: Nat Commun. 2018 Oct 30;9:4522. doi: 10.1038/s41467-018-06790-1 (PMC6207713; doi:10.1038/s41467-018-06790-1)
Supplement: Supplementary file 3 — Description of Additional Supplementary Files [file 41467_2018_6790_MOESM3_ESM.pdf]

Supplementary Data File Legends:

Supplementary Data 1 | Mass spectrometry derived peptides identified from recombinant Src incubated with various concentrations of H_2O_2 in the presence of 1.0 mM dimedone.

Supplementary Data 2 | Mass spectrometry derived peptides identified from recombinant Src incubated with various concentrations of H_2O_2 in the presence of 100 µM BCN.

Supplementary Data 3 | Mass spectrometry derived peptides identified from recombinant Src incubated with 0.2 mM H_2O_2 in the presence of 1.0 mM NBD-Cl.

Supplementary Data 4 | Summary of targeted mass spectrometry with PRM for quantitative analysis of dimedone-labeled peptides identified during quantitative analysis using light and heavy dimedone (dim/dim-d6) labeling.

Supplementary Data 5 | MS/MS spectra of all the cysteine containing peptides with dimedone/dimedone-d6 modification acquired during targeted mass spectrometry with PRM.

Supplementary Movie File Legends:

Supplementary Movie 1 | MD simulation of fully reduced autoinhibited Src (Cys-277-SH, Cys-185-SH) showing minimal structural alterations of the activation loop α-helix.

Supplementary Movie 2 | MD simulation of the sulfenylated autoinhibited Src (Cys-277-SOH, Cys-185-SH) showing unfolding of the activation loop α-helix.

Supplementary Movie 3 | MD simulation of fully reduced autoinhibited Src (Cys-277-SH, Cys-185-SH) showing the SH2 domain and C-terminal tail (pY527) interface.

Supplementary Movie 4 | MD simulation of sulfenylated autoinhibited Src (Cys-277-SH, Cys-185-SOH) showing the SH2 domain and C-terminal tail (pY527) interface.
