# Supplementary material for: Direct cysteine sulfenylation drives activation of the Src kinase
Source: Nat Commun. 2018 Oct 30;9:4522. doi: 10.1038/s41467-018-06790-1 (PMC6207713; doi:10.1038/s41467-018-06790-1)
Supplement: Supplementary file 8 — Supplementary Data 5 [file 41467_2018_6790_MOESM8_ESM.pdf]

Supplemental Dataset 5: MSMS spectra of all the cysteine containing peptides with dimedone/dimedone-d6 modification acquired during targeted mass spectrometry with PRM. The Xcorr values and mass error (ppm) of the measured precursor are listed for each peptide. The delta mass error (Da) and coverage of measured fragment ions are illustrated.

AANILVGENLVC(dim-d6)KVADFGLAR Xcorr: 3.03 (2+) -0.0057 ppm

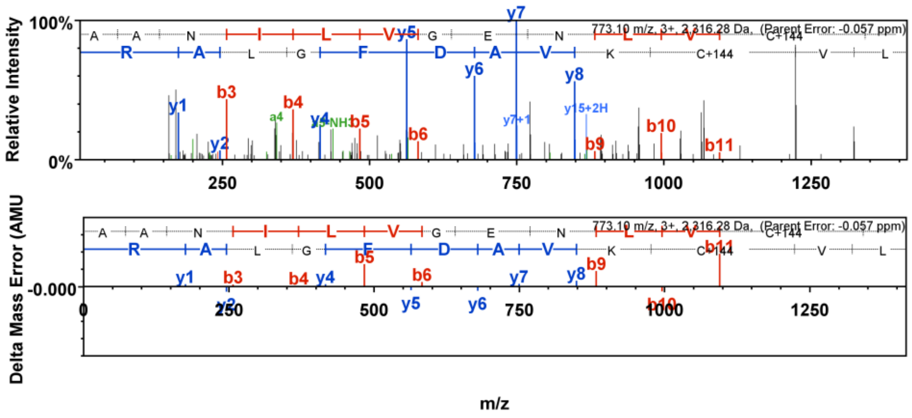

B	B Ions	B+2H	B-NH3	B-H2O	AA	Y Ions	Y+2H	Y-NH3	Y-H2O	Y
1	72.0	36.5			A	2,317.3	1,159.1	2,300.3	2,299.3	21
2	143.1	72.0			A	2,246.2	1,123.6	2,229.2	2,228.2	20
3	257.1	129.1	240.1		N	2,175.2	1,088.1	2,158.2	2,157.2	19
4	370.2	185.6	353.2		I	2,061.2	1,031.1	2,044.1	2,043.2	18
5	483.3	242.1	466.3		L	1,948.1	974.5	1,931.1	1,930.1	17
6	582.4	291.7	565.3		V	1,835.0	918.0	1,818.0	1,817.0	16
7	639.4	320.2	622.4		G	1,735.9	868.5	1,718.9	1,717.9	15
8	768.4	384.7	751.4	750.4	E	1,678.9	840.0	1,661.9	1,660.9	14
9	882.5	441.7	865.4	864.5	N	1,549.9	775.4	1,532.8	1,531.9	13
10	995.6	498.3	978.5	977.5	L	1,435.8	718.4	1,418.8	1,417.8	12
11	1,094.6	547.8	1,077.6	1,076.6	V	1,322.7	661.9	1,305.7	1,304.7	11
12	1,341.7	671.4	1,324.7	1,323.7	C+144	1,223.7	612.3	1,206.6	1,205.7	10
13	1,469.8	735.4	1,452.8	1,451.8	K	976.6	488.8	959.5	958.5	9
14	1,568.9	785.0	1,551.9	1,550.9	V	848.5	424.7	831.4	830.5	8
15	1,639.9	820.5	1,622.9	1,621.9	A	749.4	375.2	732.4	731.4	7
16	1,755.0	878.0	1,737.9	1,737.0	D	678.4	339.7	661.3	660.3	6
17	1,902.0	951.5	1,885.0	1,884.0	F	563.3	282.2	546.3		5
18	1,959.1	980.0	1,942.0	1,941.0	G	416.3	208.6	399.2		4
19	2,072.1	1,036.6	2,055.1	2,054.1	L	359.2	180.1	342.2		3
20	2,143.2	1,072.1	2,126.1	2,125.2	A	246.2	123.6	229.1		2
21	2,317.3	1,159.1	2,300.3	2,299.3	R	175.1	88.1	158.1		1

GAYC(dim-d6)LSVSDFDNAK Xcorr: 2.62 (2+) 0.0079 ppm

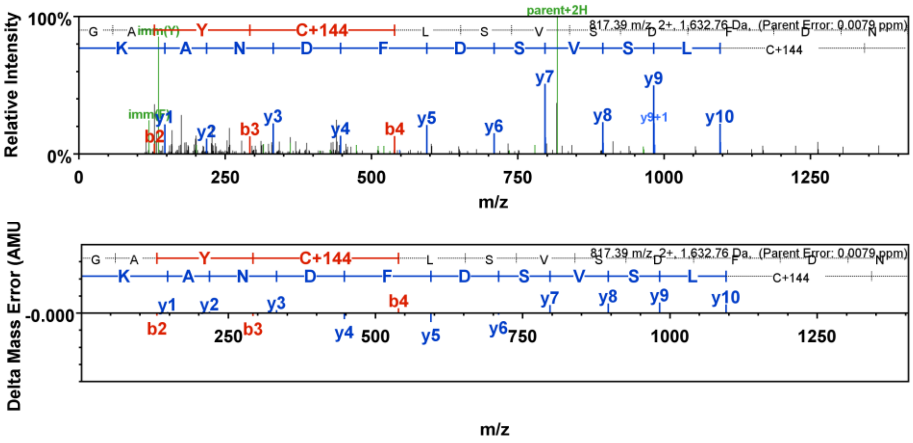

B	B Ions	B+2H	B-NH3	B-H2O	AA	Y Ions	Y+2H	Y-NH3	Y-H2O	Y
1	58.0				G	1,633.8	817.4	1,616.7	1,615.8	14
2	129.1				A	1,576.7	788.9	1,559.7	1,558.7	13
3	292.1			274.1	Y	1,505.7	753.4	1,488.7	1,487.7	12
4	539.2			521.2	C+144	1,342.6	671.8	1,325.6	1,324.6	11
5	652.3			634.3	L	1,095.5	548.3	1,078.5	1,077.5	10
6	739.4	370.2		721.3	S	982.4	491.7	965.4	964.4	9
7	838.4	419.7		820.4	V	895.4	448.2	878.4	877.4	8
8	925.5	463.2		907.5	S	796.3	398.7	779.3	778.3	7
9	1,040.5	520.7		1,022.5	D	709.3	355.2	692.3	691.3	6
10	1,187.6	594.3		1,169.5	F	594.3		577.3	576.3	5
11	1,302.6	651.8		1,284.6	D	447.2		430.2	429.2	4
12	1,416.6	708.8	1,399.6	1,398.6	N	332.2		315.2	314.2	3
13	1,487.7	744.3	1,470.6	1,469.7	A	218.1		201.1		2
14	1,633.8	817.4	1,616.7	1,615.8	K	147.1		130.1		1

HADGLC(dim-d6)HR Xcorr: 3.34 (3+) 0.12 ppm

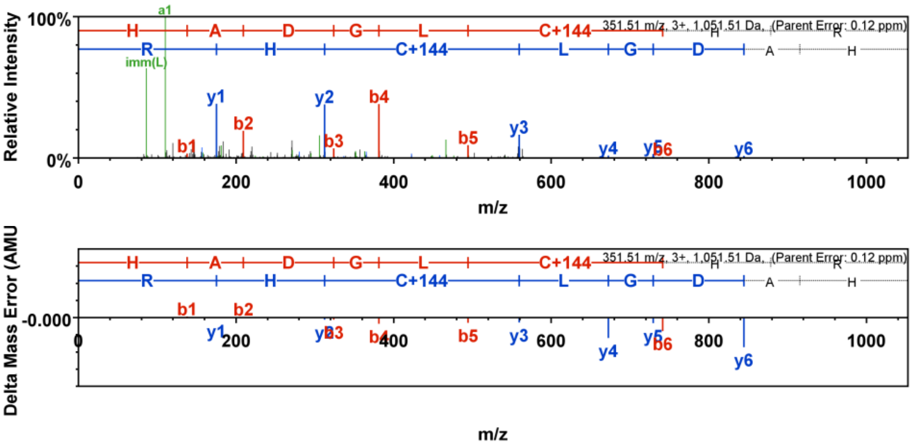

B	B Ions	B+2H	B-NH3	B-H2O	AA	Y Ions	Y+2H	Y-NH3	Y-H2O	Y
1	138.1	69.5			H	1,052.5	526.8	1,035.5	1,034.5	8
2	209.1	105.1			A	915.5	458.2	898.4	897.5	7
3	324.1	162.6		306.1	D	844.4	422.7	827.4	826.4	6
4	381.2	191.1		363.1	G	729.4	365.2	712.4		5
5	494.2	247.6		476.2	L	672.4	336.7	655.4		4
6	741.4	371.2		723.3	C+144	559.3	280.2	542.3		3
7	878.4	439.7		860.4	H	312.2	156.6	295.2		2
8	1,052.5	526.8	1,035.5	1,034.5	R	175.1	88.1	158.1		1

LGQGC(dim-d6)FGEVWMGTWNGTTR Xcorr: 5.10 (2+)   -0.42 ppm

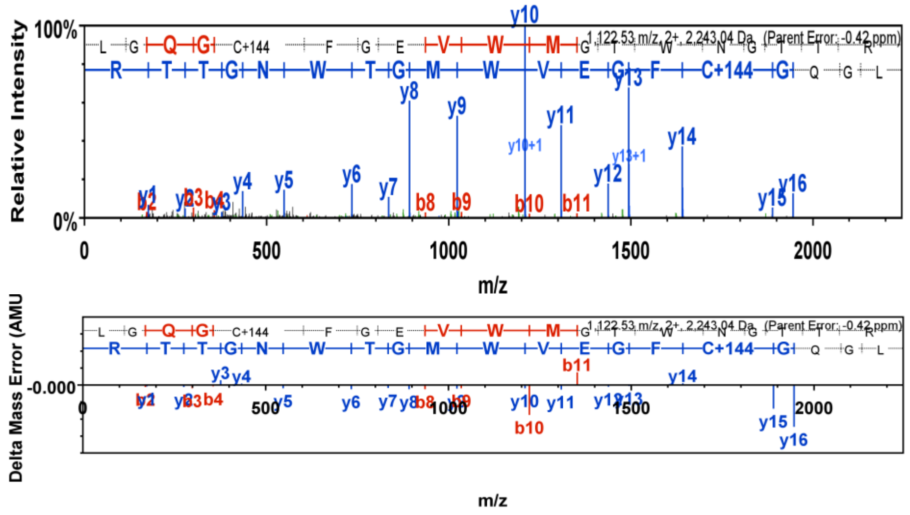

B	B Ions	B+2H	B-NH3	B-H2O	AA	Y Ions	Y+2H	Y-NH3	Y-H2O	Y
1	114.1				L	2,244.0	1,122.5	2,227.0	2,226.0	19
2	171.1				G	2,131.0	1,066.0	2,113.9	2,113.0	18
3	299.2		282.1		Q	2,073.9	1,037.5	2,056.9	2,055.9	17
4	356.2		339.2		G	1,945.9	973.4	1,928.9	1,927.9	16
5	603.3		586.3		C+144	1,888.9	944.9	1,871.8	1,870.9	15
6	750.4	375.7	733.3		F	1,641.7	821.4	1,624.7	1,623.7	14
7	807.4	404.2	790.4		G	1,494.7	747.8	1,477.7	1,476.7	13
8	936.4	468.7	919.4	918.4	E	1,437.7	719.3	1,420.6	1,419.6	12
9	1,035.5	518.3	1,018.5	1,017.5	V	1,308.6	654.8	1,291.6	1,290.6	11
10	1,221.6	611.3	1,204.6	1,203.6	W	1,209.5	605.3	1,192.5	1,191.5	10
11	1,352.6	676.8	1,335.6	1,334.6	M	1,023.5	512.2	1,006.4	1,005.5	9
12	1,409.7	705.3	1,392.6	1,391.6	G	892.4	446.7	875.4	874.4	8
13	1,510.7	755.9	1,493.7	1,492.7	T	835.4	418.2	818.4	817.4	7
14	1,696.8	848.9	1,679.8	1,678.8	W	734.4	367.7	717.3	716.3	6
15	1,810.8	905.9	1,793.8	1,792.8	N	548.3		531.3	530.3	5
16	1,867.8	934.4	1,850.8	1,849.8	G	434.2		417.2	416.2	4
17	1,968.9	984.9	1,951.9	1,950.9	T	377.2		360.2	359.2	3
18	2,069.9	1,035.5	2,052.9	2,051.9	T	276.2		259.1	258.2	2
19	2,244.0	1,122.5	2,227.0	2,226.0	R	175.1		158.1		1

LTTVC(dim-d6)PTSKPQTQGLAK   Xcorr: 3.38 (3+)   -0.14 ppm

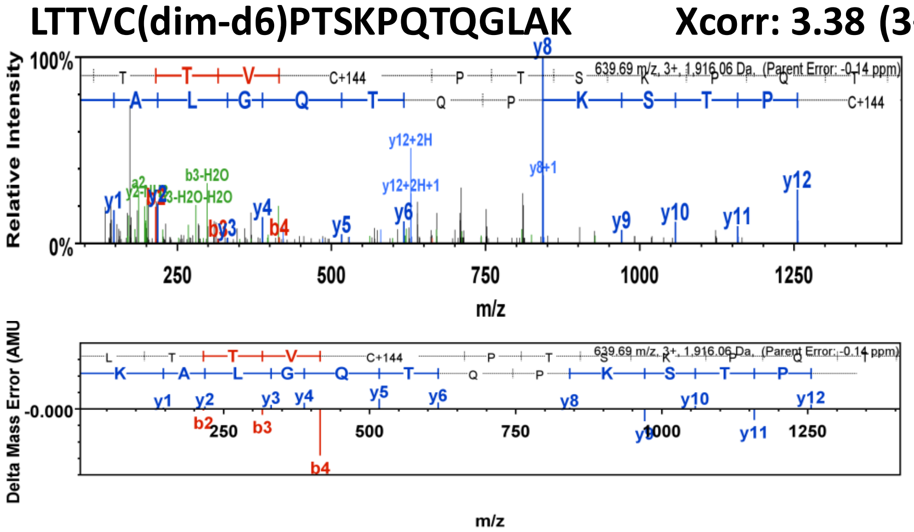

B	B Ions	B+2H	B-NH3	B-H2O	AA	Y Ions	Y+2H	Y-NH3	Y-H2O	Y
1	114.1	57.5			L	1,917.1	959.0	1,900.0	1,899.1	17
2	215.1	108.1		197.1	T	1,804.0	902.5	1,787.0	1,786.0	16
3	316.2	158.6		298.2	T	1,702.9	852.0	1,685.9	1,684.9	15
4	415.3	208.1		397.2	V	1,601.9	801.4	1,584.9	1,583.9	14
5	662.4	331.7		644.4	C+144	1,502.8	751.9	1,485.8	1,484.8	13
6	759.4	380.2		741.4	P	1,255.7	628.4	1,238.7	1,237.7	12
7	860.5	430.7		842.5	T	1,158.6	579.8	1,141.6	1,140.6	11
8	947.5	474.3		929.5	S	1,057.6	529.3	1,040.6	1,039.6	10
9	1,075.6	538.3	1,058.6	1,057.6	K	970.6	485.8	953.5	952.6	9
10	1,172.7	586.8	1,155.6	1,154.6	P	842.5	421.7	825.4	824.5	8
11	1,300.7	650.9	1,283.7	1,282.7	Q	745.4	373.2	728.4	727.4	7
12	1,401.8	701.4	1,384.7	1,383.7	T	617.4	309.2	600.3	599.4	6
13	1,529.8	765.4	1,512.8	1,511.8	Q	516.3	258.7	499.3		5
14	1,586.8	793.9	1,569.8	1,568.8	G	388.3	194.6	371.2		4
15	1,699.9	850.5	1,682.9	1,681.9	L	331.2	166.1	314.2		3
16	1,771.0	886.0	1,753.9	1,752.9	A	218.1	109.6	201.1		2
17	1,917.1	959.0	1,900.0	1,899.1	K	147.1	74.1	130.1		1

MPC(dim-d6)PPEPESLHDLMcQcWR Xcorr: 3.22 (2+)   -0.11 ppm

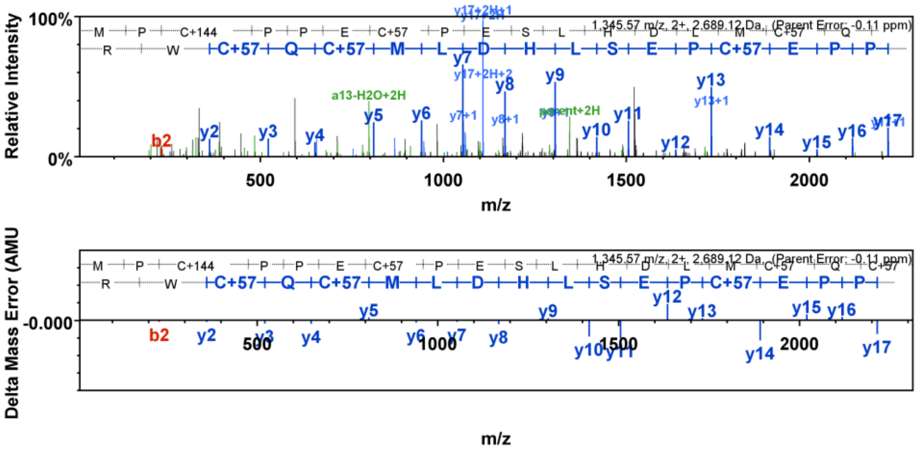

B	B Ions	B+2H	B-NH3	B-H2O	AA	Y Ions	Y+2H	Y-NH3	Y-H2O	Y
1	132.0				M	2,690.1	1,345.6	2,673.1	2,672.1	20
2	229.1				P	2,559.1	1,280.0	2,542.1	2,541.1	19
3	476.2				C+144	2,462.0	1,231.5	2,445.0	2,444.0	18
4	573.3				P	2,214.9	1,108.0	2,197.9	2,196.9	17
5	670.3				P	2,117.9	1,059.4	2,100.8	2,099.9	16
6	799.4	400.2		781.4	E	2,020.8	1,010.9	2,003.8	2,002.8	15
7	959.4	480.2		941.4	C+57	1,891.8	946.4	1,874.7	1,873.8	14
8	1,056.4	528.7		1,038.4	P	1,731.7	866.4	1,714.7	1,713.7	13
9	1,185.5	593.2		1,167.5	E	1,634.7	817.8	1,617.7	1,616.7	12
10	1,272.5	636.8		1,254.5	S	1,505.6	753.3	1,488.6	1,487.6	11
11	1,385.6	693.3		1,367.6	L	1,418.6	709.8	1,401.6	1,400.6	10
12	1,522.7	761.8		1,504.7	H	1,305.5	653.3	1,288.5	1,287.5	9
13	1,637.7	819.3		1,619.7	D	1,168.5	584.7	1,151.4	1,150.5	8
14	1,750.8	875.9		1,732.8	L	1,053.4	527.2	1,036.4		7
15	1,881.8	941.4		1,863.8	M	940.4	470.7	923.3		6
16	2,041.8	1,021.4		2,023.8	C+57	809.3		792.3		5
17	2,169.9	1,085.5	2,152.9	2,151.9	Q	649.3		632.3		4
18	2,329.9	1,165.5	2,312.9	2,311.9	C+57	521.2		504.2		3
19	2,516.0	1,258.5	2,499.0	2,498.0	W	361.2		344.2		2
20	2,690.1	1,345.6	2,673.1	2,672.1	R	175.1		158.1		1
